# Correction: Anticipatory changes in British household purchases of soft drinks associated with the announcement of the Soft Drinks Industry Levy: A controlled interrupted time series analysis

**DOI:** 10.1371/journal.pmed.1004201

**Published:** 2023-03-13

**Authors:** Nina T. Rogers, David Pell, Tarra L. Penney, Oliver Mytton, Adam Briggs, Steven Cummins, Mike Rayner, Harry Rutter, Peter Scarborough, Stephen J. Sharp, Richard D. Smith, Martin White, Jean Adams

This article [1] was republished on February 13^th^, 2023, to incorporate revised data analyses (discussed below) and update the author list and funding statement. Please download this article again to view the correct version. The originally published, uncorrected article and the republished, corrected articles are provided here for reference.

## Data analysis updates

In the process of conducting additional analyses exploring the impact of the UK Soft Drinks Industry Levy (SDIL) on purchasing of drinks and confectionary around 2 years post implementation, we identified an error involving a weighting variable that was incorporated into the data analyses reported in [1].

One important use of weighting variables is to take account of the fact that the people who take part in surveys and other research do not always reflect the make-up of the whole population. For instance, women are often more likely than men to take part in research. Weighting variables can correct for these differences. They are widely used in health research.

The data used in these papers is an extract from the Kantar WorldPanel household purchasing panel (http://www.kantarworldpanel.com/). Households in the panel provide information on all grocery purchases brought home. Our data extract includes purchases of drinks, confectionary and toiletries but excludes all other purchases. Our analyses focus on changes in weekly purchases of drinks in different categories over time in relation to announcement and implementation of the UK Soft Drinks Industry Levy (SDIL), as well as of confectionary and toiletries.

Two weighting variables were used in the analyses reported in the original published version of this article [1]. Firstly, a design weight provided by Kantar takes account of who responds to the survey and is applied at the level of individual purchases to make the data more representative of Great Britain. Secondly, a household weekly weight was calculated by us to take account of possible differences in the number of households in Great Britain.

Following detailed exploration, we concluded that the household weekly weight was miscalculated. Further, additional discussion with Kantar has confirmed that no additional household weekly weight is required and any changes in the household structure of Great Britain is taken into account in the design weight.

In the updated version of the article the data were reanalyzed to exclude the household weekly weighting variable. Removal of this variable is the only change made to the analysis. The updated analyses were reviewed and approved by *PLOS Medicine*, with the caveat that neither the primary data nor aggregate data underlying the analyses were available to the Editors or statistical reviewer.

## Impact of the error on results

As the erroneous household weekly weighting variable was used throughout the original analysis, removing it has affected all results–some by a negligible amount, others more substantively.

## Impact of error on conclusions

This article [1] reports on changes in household purchasing in the two years following announcement of the SDIL in 2016. We studied trends in purchasing from two years before the announcement to two years after the announcement, but before implementation (i.e. from 2014 to 2018).

Based on analyses including the household weekly weighting variable, the original version of the article reported no statistically significant change in total volume of, or sugar from, soft drinks purchased. The reanalyses likewise did not indicate a statistically significant change in total *volume* of soft drinks purchased. However, when the household weekly weighting variable was excluded from the analyses we found a statistically significant increase in *sugar purchased from soft drinks* of 5.3g per household per week or 1.7%.

The study’s original conclusion was that “the announcement of the levy was associated with reductions in volume and sugar purchased in lower-levy-tier drinks [those with 5-8g sugar per 100ml] before implementation. These were offset by increases in sugar purchased from no-levy drinks [those with less than 5g sugar per 100ml].”

The revised conclusion is that “the announcement of the levy was associated with reductions in volume and sugar purchased in lower levy tier drinks before implementation. These were offset by increases in purchasing of higher-levy [those with more than 8g sugar per 100ml] and no levy drinks.” We interpret our findings as potentially reflecting “reformulation of drinks from the lower to no levy tier with removal of some, but not all, sugar, alongside changes in consumer attitudes, beliefs and purchasing behaviours.”

In other words, whilst the total volume of soft drinks did not seem to change following the announcement of the levy, there was a small increase in the amount of sugar households purchased in soft drinks. This means that at the point just before the levy was implemented, additional action (such as implementation of the levy) was required to achieve benefits to public health.

## Additional limitations of this study

Aspects of the study methods are proprietary and were not available to the authors, editors, and reviewers. This includes whether/how data were cleaned or pre-processed before analyses reported in the *PLOS Medicine* article, details of how the design weight was calculated, and the “undisclosed propriety minimum value” in weekly household spending that was used as an exclusion criterion.

## Future work

Our evaluation of the SDIL is ongoing. We have further work in process examining changes in purchasing around 2 years after implementation. We are also exploring how the SDIL impacted on purchasing differently in different households–according to their income and whether or not they included children. Other work is exploring the impacts of the SDIL on obesity in children, on dental health outcomes, on total diet, on longer term health in adults, and on the economy.

## Publisher’s Note

As is indicated in the article’s Data Availability Statement [1], this study used proprietary data owned by Kantar Worldpanel, and interested readers should contact the data owner directly to request access. The primary data were not available to the editors or reviewers during pre-publication peer review or the post-publication evaluation of the data analysis errors and revisions.

Due to this issue and the proprietary nature of some methodological details, *PLOS Medicine* has been limited in our ability to evaluate the data analyses and whether methods of data collection or processing impact the validity and generalizability of the article’s results and conclusions.

## Supporting information

S1 File Originally published, uncorrected article. (PDF)

S2 File Republished, corrected article. (PDF)
