# Supplementary figures and images for: Anticipatory changes in British household purchases of soft drinks associated with the announcement of the Soft Drinks Industry Levy: A controlled interrupted time series analysis
Source: PLoS Med. 2020 Nov 12;17(11):e1003269. doi: 10.1371/journal.pmed.1003269 (PMC7660521; doi:10.1371/journal.pmed.1003269)

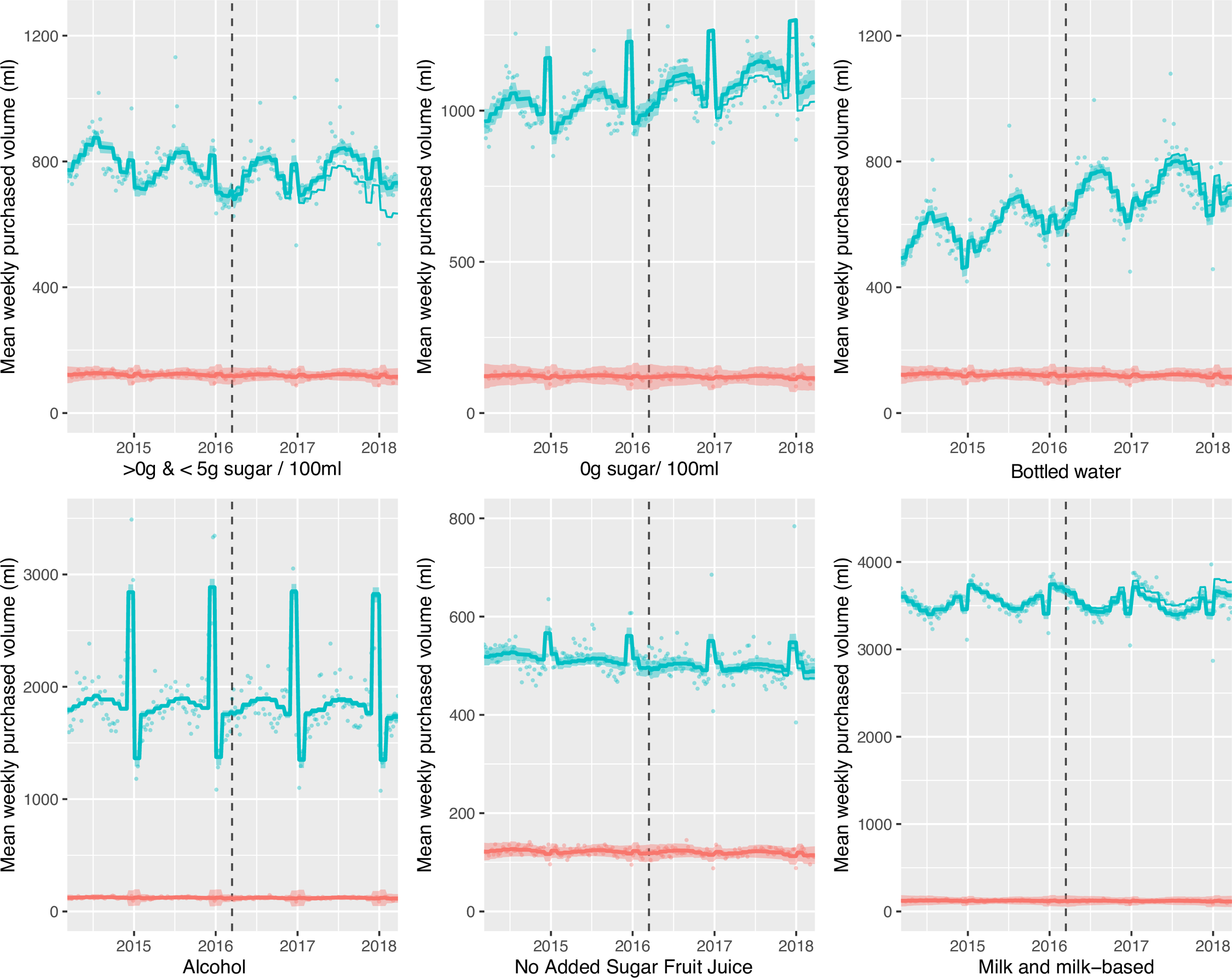

Supplement: S1 Fig — Notes. Points are observed data, thick lines (with shadows) are modelled data (and 95% confidence intervals); thin lines (without shadows) are the counterfactual had the announcement not happened; blue points and lines are drinks; red points and lines are the control category of toiletries; the vertical dashed line indicates the point of announcement of the SDIL; Y-axes vary in scale between panels to maximise the resolution of figures; modelled purchases include all adjustments as described in the methods section. (TIF) [file pmed.1003269.s002.tif]

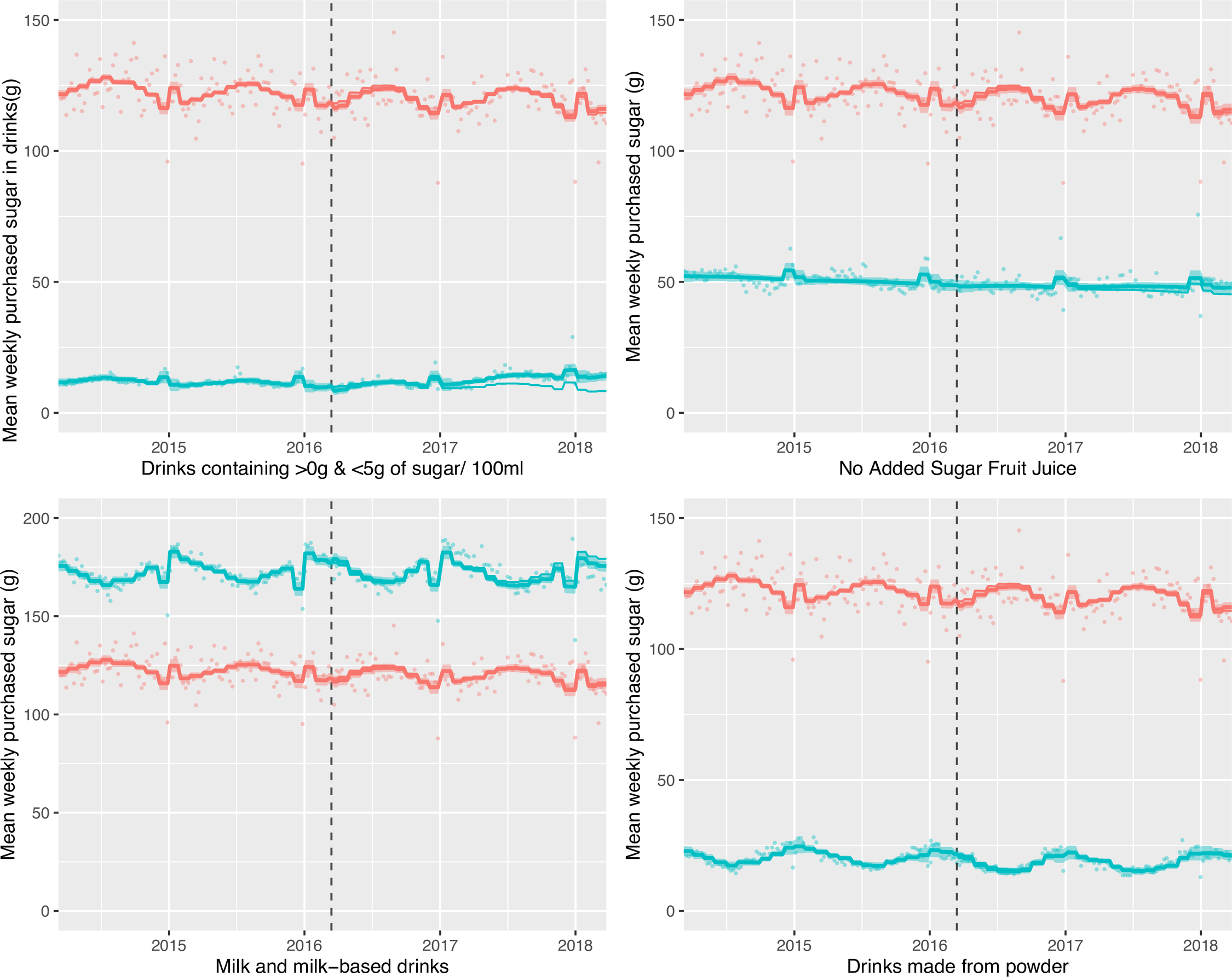

Supplement: S2 Fig — Notes. Points are observed data, thick lines (with shadows) are modelled data (and 95% confidence intervals); thin lines (without shadows) are the counterfactual had the announcement not happened; blue points and lines are drinks; red points and lines are the control category of toiletries; the vertical dashed line indicates the point of announcement of the SDIL; Y-axes vary in scale between panels to maximise the resolution of figures; modelled purchases include all adjustments as described in the methods section. (TIF) [file pmed.1003269.s003.tif]

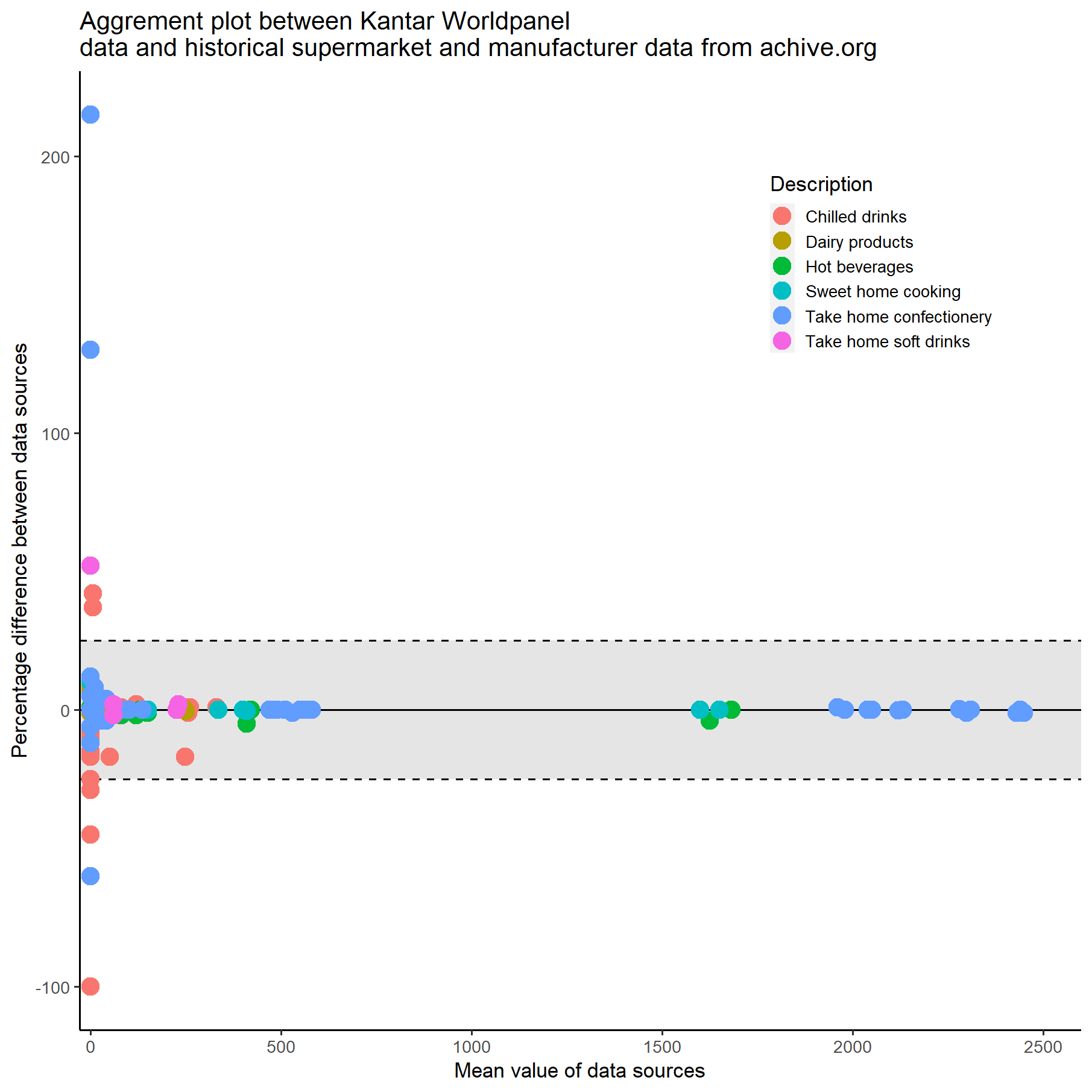

Supplement: S3 Fig — (TIF) [file pmed.1003269.s004.tif]
