# Supplementary material for: Anticipatory changes in British household purchases of soft drinks associated with the announcement of the Soft Drinks Industry Levy: A controlled interrupted time series analysis
Source: PLoS Med. 2020 Nov 12;17(11):e1003269. doi: 10.1371/journal.pmed.1003269 (PMC7660521; doi:10.1371/journal.pmed.1003269)
Supplement: S1 Text — (DOCX) [file pmed.1003269.s005.docx]

**S1 Text:**

S1 Table. Demographic characteristics of Kantar Worldpanel households from March 2014 – March 2018 (weighted)

| Characteristic | | Kantar Worldpanel (%) | UK population |
| --- | --- | --- | --- |
| Children in household^1^ | |  |  |
|  | No | 65.3 | 71.5 |
|  | Yes | 34.6 | 28.5 |
| Social grade of chief income earner^2^ | |  |  |
|  | AB: Higher and intermediate managerial | 19.3 | 27 |
|  | C1: Junior managerial | 34.1 | 28 |
|  | C2: Skilled manual workers | 16.4 | 20 |
|  | D: Semi and unskilled-manual workers | 12.4 | 15 |
|  | E: lowest grade workers | 7.9 | 10 |
| Total household income (£ per annum)^3^ | |  |  |
|  | 0-9,999 | 6.4 |  |
|  | 10,000-19,999 | 18.9 |  |
|  | 20,000-29,999 | 17.2 |  |
|  | 30,000-39,999 | 13.1 |  |
|  | 40,000-49,999 | 9.0 |  |
|  | 50,000-59,999 | 5.5 |  |
|  | 60,000-69,999 | 2.9 |  |
|  | 70,000+ | 4.1 |  |
|  | Refused to answer | 14.4 |  |
|  | Mean (£) |  | 33,264 |
|  | Median (£) |  | 27,963 |
| Highest qualification of chief income earner^4^ | |  |  |
|  | Higher than School leaving qualifications taken at ~18 years (e.g. A-Levels) | 16.2 | 43.8 |
|  | School leaving qualifications taken at ~18 years (e.g. A-Levels) | 65.3 | 22.4 |
|  | School leaving qualifications taken at ~16 years (e.g. GCSE) | 34.6 | 18.7 |
|  | Other (including no qualifications and unknown) | 19.3 | 15.1 |

^1^UK population figure is average of households with dependent children from 2014-2018

^2^UK population figures from 2016

^3^No directly comparable figures available from ONS, mean and medians are averaged over 2014-2018

^4^UK population figures from 2014
