# Supplementary material for: Anticipatory changes in British household purchases of soft drinks associated with the announcement of the Soft Drinks Industry Levy: A controlled interrupted time series analysis
Source: PLoS Med. 2020 Nov 12;17(11):e1003269. doi: 10.1371/journal.pmed.1003269 (PMC7660521; doi:10.1371/journal.pmed.1003269)
Supplement: S2 Text — (DOCX) [file pmed.1003269.s006.docx]

***S2 Text:***

***Sensitivity analysis 1: Exemptions for small manufacturers and producers***

In sensitivity analysis 1 average annual sales of levy liable drinks was estimated from our data, and manufacturers with <1M or <0.5M litres of liable product were excluded from the analysis. Controlled ITS analysis was carried out for both levy liable tiers and the results are very similar to those in the main analyses (Table 2 and Table 3) where all manufacturers were included.

For example, in the main analysis (Table 2 and Table 3), after 2 years we found a relative increase in purchase volume of higher tier drinks of 9.1% and a relative decrease in purchase volume of lower tier drinks of 38.4%. Comparable figures for sugar were 10.2% and -38.2%.

When manufacturers with sales of <1M litres per year of liable product were excluded the figures were 9.5%, -39.0%, 11.4% and -41.6%; when manufacturers with sales of <0.5M litres per year of liable product were excluded the figures were 9.6%, -39.0%, 9.8% and -41.6% (see S2 Table and S3 Table).

S2 Table Adjusted change in mean volume of drinks (ml) purchased per household per week (95% CI) (level) and adjusted change per week (trend) post-announcement of the Soft Drinks Industry Levy including toiletries as a control condition, with absolute and relative differences in purchased volume at two-years post-announcement; excluding small manufacturers

|  | |  |  | Change at 2 years after the SDIL announcement | |
| --- | --- | --- | --- | --- | --- |
| Category | | Level change (ml) | Trend change (ml per week) | Absolute change (ml) | Relative change (%) |
| <1 M Litres | |  |  |  |  |
|  | Higher tier ≥8g of sugar per 100ml | **48.8 (8.4, 89.2)** | -0.1 (-0.7, 0.5) | **42.2 (20.3, 64.1)** | **9.5 (4.6, 14.4)** |
|  | Lower tier ≥5g - <8g of sugar per 100ml | -2.1 (-25.1, 21.0) | **-0.66 (-1.01, -0.3)** | **-68.9 (-82.1, -55.8)** | **-39.0 (-46.5, -31.6)** |
| <0.5M Litres | |  |  |  |  |
|  | Higher tier ≥8g of sugar per 100ml | **48.2 (7.6, 88.8)** | -0.08 (-0.7, 0.5) | **42.8 (20.8, 64.9)** | **9.6 (4.7, 14.5)** |
|  | Lower tier ≥5g - <8g of sugar per 100ml | -2.1 (-25.1, 21.8) | **-0.7 (-1.1, -0.2)** | **-69.0 (-82.1, -55.9)** | **-39.0 (-46.4, -31.6)** |

Level change is the difference between the model estimates and the counterfactual at the first week after the SDIL announcement. The trend change is the mean change in the slope of purchases following the announcement. The absolute and relative differences represent the difference between the counterfactual and the model estimates in the final week of the study. Estimates statistically significant at the p<0.05 level are highlighted in bold

S3 Table Adjusted change in mean sugar (g) in drinks purchased per household per week (95% CI) (level) and adjusted change per week (trend) post-announcement of the Soft Drinks Industry Levy, with absolute and relative differences in purchased volume at two-years post-announcement; excluding small manufacturers

|  | |  |  |  | Change at 2 years after the SDIL announcement | |
| --- | --- | --- | --- | --- | --- | --- |
| Category | |  | Level change (g) | Trend change (g per week) | Absolute change (g) | Relative change (%) |
| <1 M Litres | |  | |  |  |  |
|  | Higher tier ≥8g of sugar per 100ml | **5.1 (0.4, 9.8)** | | -0.03 (-0.1 ,0.04) | **5.5 (2.9, 8.1)** | **11.4 (6.1, 16.7)** |
|  | Lower tier ≥5g - <8g of sugar per 100ml^†^ | -0.7 (-2.5, 1.0) | | **-0.07 (-0.1, -0.03)** | **-4.9 (-3.9, -6.0)** | **-41.6 (-50.6, -32.6)** |
| <0.5M Litres | |  | |  |  |  |
|  | Higher tier ≥8g of sugar per 100ml | **4.9 (0.2, 9.7)** | | -0.03 (-0.1, 0.04) | **4.9 (2.3,7.4)** | **9.8 (4.6, 15.0)** |
|  | Lower tier ≥5g - <8g of sugar per 100ml^†^ | -0.7 (-2.5, 1.0) | | **-0.07 (-0.1, -0.03)** | **-4.9 (-3.9, -6.0)** | **-41.6 (-50.6, -32.7)** |

Level change is the difference between the model estimates and the counterfactual at the first week after the SDIL announcement. The trend change is the mean change in the slope of purchases following the announcement. The absolute and relative differences represent the difference between the counterfactual and the model estimates in the final week of the study. †Trend^2^ indicates the sugar change multiplied by weeks since the announcement squared; estimates statistically significant at the p<0.05 level are highlighted in bold
