# Supplementary material for: Anticipatory changes in British household purchases of soft drinks associated with the announcement of the Soft Drinks Industry Levy: A controlled interrupted time series analysis
Source: PLoS Med. 2020 Nov 12;17(11):e1003269. doi: 10.1371/journal.pmed.1003269 (PMC7660521; doi:10.1371/journal.pmed.1003269)
Supplement: S3 Text — (DOCX) [file pmed.1003269.s007.docx]

***S3 Text:***

***Sensitivity analysis 2: Combining drinks categories***

Many drinks with relatively high levels of sugar are excluded from the levy (e.g. fruit juices and milk-based drinks). In sensitivity analysis 2, we combined all soft drinks (irrespective of levy liability) both according to the sugar cut-offs in the levy and altogether.

When drinks were classified by their sugar content, regardless of SDIL liability, effect sizes seen in the main analyses were attenuated, but the direction and significance of effects remained unchanged. For example, in the main analysis (Table 2 and Table 3), after 2 years we found a relative increase in purchase volume of and sugar from drinks with ≥8g sugar per 100ml (higher tier) of 9.1% (95% CI: 4.1, 14.0) and 10.2% (95% CI: 4.0, 16.4) respectively. Comparable figures when all drinks were combined (S4 Table and S5 Table) were 8.2% (95% CI: 5.2, 11.1) and 8.3% (95% CI: 5.6, 11.1). The largest change were seen with respect to drinks with <5g sugar per 100ml (no levy). Here the main analysis identified an increase in purchase volume of and sugar from drinks of 10.0% (95% CI: 6.0, 13.9) and 68.5% (95% CI: 48.6, 88.4). When all drinks were combined, these effects reduced to 2.0% (95% CI: 0.05, 3.9) and 3.7% (95% CI: 1.4, 5.8).

When the volume and sugar content of all drinks combined was examined, compared to the counterfactual scenario there were no changes in purchased volume of drinks but a small increase in purchased sugar from drinks following the announcement of the levy of 5.3g (95% CI: 1.2, 9.4) or 1.7% (95% CI: 0.4, 3.0).

S4 Table Adjusted change in mean volume (ml) of all non-alcoholic drinks purchased per household per week (95% CI) (level) and adjusted change per week (trend) post-announcement of the Soft Drinks Industry Levy, including toiletries as a control condition, with absolute and relative differences in purchased volume at two-years post-announcement

|  |  |  | Change at 2 years after the SDIL announcement | |
| --- | --- | --- | --- | --- |
| Category | Level change (ml) | Trend change (ml per week) | Absolute change (ml) | Relative change (%) |
| All drinks containing ≥8g of sugar per 100ml | 29.8 (-24.1, 83.8) | 0.5 (-0.4, 1.3) | **81.4 (52.2, 110.5)** | **8.2 (5.2, 11.1)** |
| All drinks containing ≥5g - <8g of sugar per 100ml | 14.5 (-58.6, 87.6) | 0.05 (-3.1, 3.2) | **-325.7 (-414.5, -236.9)** | **-28.6 (-36.4, -20.8)** |
| All drinks containing <5g of sugar per 100ml | -131.5 (-56.1, 319.1) | 2.2 (-0.9,5.4) | **105.1 (2.9, 207.4)** | **2.0 (0.05, 3.9)** |
| All drinks | 56.5 (-143.6, 256.6) | -0.5 (-3.7, 2.7) | 4.8 (-103.5, 113.1) | 0.07 (-1.4,.1.6) |

Level change is the difference between the model estimates and the counterfactual at the first week after the SDIL announcement. The trend change is the mean change in the slope of purchases following the announcement. The absolute and relative differences represent the difference between the counterfactual and the model estimates in the final week of the study. Estimates statistically significant at the p<0.05 level are highlighted in bold

S5 Table Adjusted change in mean sugar (g) in all non-alcoholic drinks purchased per household per week (95% CI) (level) and adjusted change per week (trend) post-announcement of the Soft Drinks Industry Levy, with absolute and relative differences in purchased volume at two-years post-announcement

|  |  |  | Change at 2 years after the SDIL announcement | |
| --- | --- | --- | --- | --- |
| Category | Level change (g) | Trend change (g per week) | Absolute change (g) | Relative change (%) |
| All drinks containing ≥8g of sugar per 100ml | 4.8 (-1.4, 11.0) | 0.04 (-0.06, 0.1) | **10.1 (6.7,13.4)** | **8.3 (5.6, 11.1)** |
| All drinks containing ≥5g - <8g of sugar per 100ml^†^ | **11.3 (5.8, 16.8)** | **-0.2 (-0.3, -0.1)** | **-9.8 (-6.8, -12.7)** | **-18.8 (-13.1, -24.5)** |
| All drinks containing <5g of sugar per 100ml^†^ | **-6.8 (-12.6, -1.0)** | **0.1 (0.01, 0.2)** | **5.1 (1.9, 8.2)** | **3.6 (1.4, 5.8)** |
| All drinks | 5.9 (-1.6, 13.4) | -0.01 (-0.1, 0.1) | **5.3 (1.2, 9.4)** | **1.7 (0.4, 3.0)** |

Level change is the difference between the model estimates and the counterfactual at the first week after the SDIL announcement. The trend change is the mean change in the slope of purchases following the announcement. The absolute and relative differences represent the difference between the counterfactual and the model estimates in the final week of the study. Estimates statistically significant at the p<0.05 level are highlighted in bold
