# Supplementary material for: Anticipatory changes in British household purchases of soft drinks associated with the announcement of the Soft Drinks Industry Levy: A controlled interrupted time series analysis
Source: PLoS Med. 2020 Nov 12;17(11):e1003269. doi: 10.1371/journal.pmed.1003269 (PMC7660521; doi:10.1371/journal.pmed.1003269)
Supplement: S4 Text — (DOCX) [file pmed.1003269.s008.docx]

***S4 Text***

***Sensitivity analysis 3: Uncontrolled interrupted time series analysis***

Interrupted time series analysis estimating drinks and confectionery purchases with and without purchases of toiletries as a control condition was carried out. The level and trend changes following the announcement are presented for purchased volumes (S6 Table) and sugar (S7 Table) below. Overall there was little difference between the controlled and uncontrolled models.

As we did not have access to other product categories it is not possible to state that toiletries were the best possible control condition though in general results are similar for the uncontrolled and controlled analyses.

S6 Table Controlled and uncontrolled interrupted time series analysis of average weekly purchased volumes of drinks and confectionery from March 2014 to March 2018 using Kantar Worldpanel data

| Category | Estimate | Control included | | No controls | |
| --- | --- | --- | --- | --- | --- |
|  |  | Effect size | 95% confidence intervals | Effect size | 95% confidence intervals |
| Higher tier (≥8g sugar per 100ml) | Level change | **51.3** | **10.1, 92.5** | **87.0** | **21.1, 152.9** |
|  | Trend change | -0.10 | -0.8, 0.6 | **-1.4** | **-2.4, -0.4** |
| Lower tier (≥5g - <8g sugar per 100ml) | Level change | 0.2 | -0.5, 0.8 | -1.9 | -25.2, 21.5 |
|  | Trend change | **-0.6** | **-1.1, -0.2** | **-0.6** | **-1.1, -0.2** |
| No levy (<5g sugar per 100ml) | Level change | -13.8 | -132.7, 105.1 | -17.4 | -110.2, 75.3 |
|  | Trend change | 1.7 | -0.4, 3.7 | **1.7** | **0.3, 3.2** |
| Drinks with >0g - <5g sugar per 100ml | Level change | -25.7 | -75.9, 24.5 | -28.9 | -68.4, 10.6 |
|  | Trend change | **1.2** | **0.4, 2.0** | 1.2 | -0.6, 1.8 |
| Drinks with 0g sugar per 100ml | Level change | 9.8 | -64.3, 83.9 | 11.5 | -60.6, 83.5 |
|  | Trend change | 0.5 | -0.8, 1.8 | 0.5 | -0.7, 1.7 |
| Bottled water | Level change | 16.0 | -36.7 63.7 | 13.8 | -38.9,66.5 |
|  | Trend change | -0.5 | -1.5, 0.4 | -0.5 | -1.4, 0.4 |
| Alcoholic drinks | Level change | -10.0 | -49.2, 29.2 | -12.4 | -51.6, 26.8 |
|  | Trend change | -0.09 | -0.5, 0.4 | -0.04 | -0.5, 0.4 |
| Milk and milk based drinks | Level change | 13.2 | -114.5, 88.1 | 4.5 | -86.7, 95.6 |
|  | Trend change | -1.3 | -3.5, 0.9 | **-1.01** | **-2.6, 0.5** |
| No added sugar fruit juices | Level change | -5.0 | -33.2, 23.2 | -3.9 | -29.8, 22.0 |
|  | Trend change | 0.2 | -0.4, 0.8 | 0.1 | -0.3, 0.5 |
| Drinks sold as a powder (g) | Level change | -2.0 | -9.0, 4.0 | -2.8 | -8.8, 3.2 |
|  | Trend change | -0.06 | -0.2, 0.04 | **-0.03** | **-0.1, 0.1** |
| Confectionery (g) | Level change | -6.9 | -48.3, 34.4 | -17.5 | -84.2, 49.2 |
|  | Trend change | -0.06 | -0.9, 0.8 | 0.1 | -1.1, 1.3 |

Level change is the difference between the model estimates and the counterfactual at the first week after the SDIL announcement. The trend change is the mean change in the slope of purchases following the announcement. Estimates statistically significant at the p<0.05 level are highlighted in bold

S7 Table Controlled and uncontrolled interrupted time series analysis of average weekly purchased sugar in drinks and confectionery from March 2014 to March 2018 using Kantar Worldpanel data

| Category | Estimate | Control included | | No controls | |
| --- | --- | --- | --- | --- | --- |
|  |  | Effect size | 95% CI | Effect size | 95% CI |
| Higher tier (≥8g sugar per 100ml) | Level change | **7.4** | **1.8, 13.0** | **5.6** | **0.8, 10.3** |
|  | Trend change | -0.03 | -0.1, 0.05 | -0.01 | -0.1, 0.07 |
| Lower tier (≥5g - <8g sugar per 100ml) | Level change | 1.8 | -1.4, 4.9 | -0.2 | -1.8, 1.3 |
|  | Trend change | **-0.07** | **-0.1, -0.03** | **-0.05** | **-0.07, -0.02** |
| No levy (<5g sugar per 100ml) | Level change | 0.2 | -2.7, 3.0 | **-1.7** | **-2.6,- 0.7** |
|  | Trend change | **0.04** | **0.001, 0.08** | **0.07** | **0.06, 0.08** |
| Drinks with >0g - <5g sugar per 100ml | Level change | 0.2 | -2.7, 3.0 | **-1.7** | **-2.6, -0.7** |
|  | Trend change | **0.04** | **0.001, 0.08** | 0.07 | 0.06, 0.08 |
| Milk and milk based drinks | Level change | 3.7 | -1.4, 8.9 | 1.8 | -1.6, 5.3 |
|  | Trend change | -0.08 | -0.2, -0.002 | -0.05 | -0.1, 0.01 |
| No added sugar fruit juices | Level change | 1.1 | -3.1, 5.3 | -0.5 | -3.1, 2.0 |
|  | Trend change | 0.002 | -0.06, 0.06 | 0.03 | -0.01, 0.07 |
| Drinks sold as a powder (g) | Level change | 0.8 | -2.7, 4.2 | -0.2 | -3.3, 1.0 |
|  | Trend change | -0.01 | -0.07, 0.05 | 0.01 | -0.03, 0.05 |
| Confectionery (g) | Level change | -6.9 | -48.3, 34.4 | -3.7 | -49.0, 41.6 |
|  | Trend change | -0.06 | -0.9, 0.8 | -0.03 | -1.07, 0.07 |

Level change is the difference between the model estimates and the counterfactual at the first week after the SDIL announcement. The trend change is the mean change in the slope of purchases following the announcement. The absolute and relative differences represent the difference between the counterfactual and the model estimates in the final week of the study. Estimates statistically significant at the p<0.05 level are highlighted in bold
