# Supplementary material for: Anticipatory changes in British household purchases of soft drinks associated with the announcement of the Soft Drinks Industry Levy: A controlled interrupted time series analysis
Source: PLoS Med. 2020 Nov 12;17(11):e1003269. doi: 10.1371/journal.pmed.1003269 (PMC7660521; doi:10.1371/journal.pmed.1003269)
Supplement: S5 Text — (DOCX) [file pmed.1003269.s009.docx]

***S5 Text:***

***Validity checks of data on sugar content of drinks***

In our analyses, drinks were categorised according to the sugar content provided by KWP. Our results are therefore dependent on having correctly assigned sugar values for each product. Thus we examined the extent to which the amount of sugar stated in the KWP data reflected the amount of sugar given on the product label. As the SDIL is aimed at stimulating reformulation by manufacturers and producers it is likely that the sugar content of products will change and therefore a historical record of product labels is required to examine the validity of products over time.

We carried out a validation study comparing the nutritional content of products recorded in KWP and compared them to the nutritional information listed on the “wayback machine” hosted by Archive.org, an internet archive. Since 1996, Archive.org has regularly saved copies of a variety of websites including food manufacturers and supermarkets, and has over 330bn web pages stored; information captured includes the date the website was saved. Stored web pages can be accessed by entering a URL in to the Archive.org search engine. Using the product description the nutritional information taken from products in the KWP data was compared to values recorded on websites collected by the wayback machine.

Methods

Due to the large number of purchases included in the data set it was not feasible to compare the nutritional information for all products at all time points. Instead, we chose to examine the top 10 products by number of purchases per KWP recording category and, in case popular products were more likely to be accurate due to the large number of entries, we also randomly chose a further 10 products per category. The KWP recording categories were chilled drinks; dairy products; hot beverages; Sweet home cooking; take home confectionery; and take home soft drinks giving 120 possible products.

Web checking

An internet search using the product description was conducted to find a website that contained the nutritional composition of each product. This focussed on manufacturers or producers websites along with the supermarkets: Asda; Iceland; Morrisons; Ocado; Poundland; Sainsbury’s; Tesco; Waitrose; and MySupermarket.co.uk. Once a current web page containing nutritional information was found, the URL was copied and pasted into the “wayback machine” where all matching records were examined, together with the date the webpage was captured. This date was used to match the collected nutritional information to the corresponding KWP product. In a small number of cases, we were unable to find a current webpage listing the nutritional content.

Comparison

All available nutritional information was taken from the archived web pages. This was typically energy (kJ), energy (kcal), fat, saturated fat, carbohydrate, sugars, fibre and sodium. Where salt was provided it was converted to sodium by multiplying the value by 0.4. In total 1626 values were recorded from the archived web pages and compared to contemporaneously collected KWP product values.

Inter-rater agreement between the two sources was found to be near perfect (a Cohen’s Kappa value >0.8) when comparing all nutrition information (Cohen’s Kappa 0.84, 95%CI: 0.82-0.86) and comparing sugar only (Cohen’s Kappa 0.82, 95%CI: 0.76-0.88). S3 Fig shows a Bland-Altman plot of the agreement between the two data sources. The x-axis shows the mean value from the nutritional information taken from the products listed in the KWP data set with corresponding archived data. Displayed on the y-axis is the percentage difference between the two data sources, coloured according to KWP product category.

S3 Fig Agreement between nutritional information reported by KWP and that collected from archived and current supermarket and manufacturer data collected by Archive.org

For the majority of cases the difference was close to 0%. There were a small number of products with large discrepancies that were investigated further. As a result, we discovered a change in the method of reporting Nesquik milkshake powders from “as sold” values to “as made up using semi-skimmed milk”. This change meant that both the reported sugar content decreased and the protein content increased dramatically. Other discrepancies that were found included the level of rounding reported by the website and the KWP data, that is values reported as <0.5 on a supermarket web page were reported as 0 in the KWP data. Where this occurred it led to large percentage difference between the two figures. In some cases it was difficult to find consistent nutritional data. For example, the Cadburys website contained a single listing for their giant buttons however differences in nutritional composition values were observed between giant buttons of varying size in both the collected data and the KWP data, this could potentially be due to staggered distribution of reformulated products or some other error. The KWP data were not completely without error however, there was an instance where the fibre content of one product changed to from 2.5g to 0.8g then 2.3g in the following weeks and this change was not reflected in the collected data suggesting a possible error in data entry.

Overall it appears that neither KWP data nor archived data was error free indicating that there is no ‘gold standard’ available for historical nutritional information data. However, the error present in the KWP data compared to the archived data appears to be unsystematic and does not indicate bias.
