# Supplementary material for: Anticipatory changes in British household purchases of soft drinks associated with the announcement of the Soft Drinks Industry Levy: A controlled interrupted time series analysis
Source: PLoS Med. 2020 Nov 12;17(11):e1003269. doi: 10.1371/journal.pmed.1003269 (PMC7660521; doi:10.1371/journal.pmed.1003269)
Supplement: Attachment — Submitted filename: Response to reviewers.docx [file pmed.1003269.s010.docx]

**Requests from the editors:**

Title- Please remove [ISRCTN18042742] from the title

Our response: this has been carried out.

Abstract- please combine the methods and findings into one section. The last sentence of this section should be a limitation of your study design

Abstract-please provide the name of the household purchasing panel used

Abstract- perhaps this ought to be in the background section? “The SDIL is a two tiered tax, announced in March 2016 and implemented in April 2018. Drinks with ≥8g of sugar per 100ml (higher levy tier) are taxed at £0.24 per litre, drinks with ≥5-<8g of sugar per 100ml (lower levy tier) are taxed at £0.18 per litre and drinks with <5g sugar per 100ml (no levy) are not taxed. Milk-based drinks, pure fruit juices, drinks sold as powder and drinks with >1.2% alcohol by volume are exempt”.

Our response: we have changed the abstract to give sections titled background, methods and findings and conclusion. As a result we have rewritten the abstract to improve the flow taking all of the suggestions in to account.

Data availability- please note that authors cannot be contacts for data requests. The code for analyses should be deposited in a repository and the details provided in the data availability statement.

Our response: We have adapted the statement relating sharing Kantar Worldpanel data from this paper: <https://journals.plos.org/plosmedicine/article?id=10.1371/journal.pmed.1003015> and amended the text to state the location of our code: “The statistical code for the analyses are available from https://github.com/MRC-Epid/SDILEvaluation. Kantar Worldpanel data are not publicly available but can be purchased from Kantar Worldpanel. (http://www.kantarworldpanel.com). The authors are not legally permitted to share the data used for this study, but interested parties may contact Kantar WorldPanel representative Sean Cannon to inquire about accessing this proprietary data (Sean.Cannon@kantar.com).”

References- please provide the full stop after the square brackets and when multiple references are cited, these can be included within one set of brackets for example: [2,3] or [8-10].

Our response: this has been carried out.

Please discuss and cite all recently published articles related to this research in the Introduction/Discussion sections as appropriate. Specifically, Scarborough et al in PLOS Medicine and Bandy et al in BMC Medicine must be discussed to clarify why similar findings are being reported separately.

Our response: we have added text as suggested in the introduction (lines 95 to 100) “The availability of SDIL liable soft drinks on supermarket shelves fell 19.5% almost two years after the announcement of the SDIL [26]. Whilst it has been reported that sales of levy eligible soft drinks fell by 50% from 2015-18, leading to an overall reduction in the amount of sugar in purchased soft drinks of 30% [27], this before-after study was not able to distinguish the impact of the SDIL from other trends in soft drinks purchases.”

In the discussion (lines 418 to 427) “A recent ITS of drinks available in supermarkets, rather than drinks purchased, in the UK found that the announcement of the SDIL was associated with a 20% drop in the proportion of levy eligible drinks containing greater than 5g of sugar per 100ml (the minimum sugar threshold for the lower levy tier) [26]. Scarborough et al also found evidence of ‘strategic reformulation’ with a new peak in the distribution of sugar content in drinks just below 5g per 100ml that was not previously evidenced. This is consistent with our finding of reductions in purchasing of lower levy tier drinks and increases in the sugar in, but not volume of, no levy tier drinks.

Similar to our unadjusted analysis (Table 1), a simple before-after analysis found that sales of levy eligible soft drinks fell by 50% from 2015-18, leading to an overall reduction in the amount of sugar in purchased soft drinks of 30% [27].”

At this stage, we ask that you include a short, non-technical Author Summary of your research to make findings accessible to a wide audience that includes both scientists and non-scientists. The Author Summary should immediately follow the Abstract in your revised manuscript. This text is subject to editorial change and should be distinct from the scientific abstract. Please see our author guidelines for more information: <https://journals.plos.org/plosmedicine/s/revising-your-manuscript#loc-author-summary>

Our response: as requested this has been added on page 4.

STROBE checklist: please use section and paragraph numbers, rather than page numbers. Please add the following statement, or similar, to the Methods: "This study is reported as per the Strengthening the Reporting of Observational Studies in Epidemiology STROBE guideline (S1 Checklist)."

Our response: an updated STROBE checklist with section and paragraph numbers has been added as requested.

**Comments from the reviewers:**

**Reviewer #1:**

One of the main limitations of the study is that the control group does not seem to follow the same pre-announcement trend neither the magnitudes look similar. I conclude this by looking at the graphs but the interrupted time series analyses provide actual tests by looking at the pre-announcement coefficients: the difference in the level (intercept) of the outcome variable between control and treatment categories prior to the announcement and the difference in the slope of the outcome variable between both categories prior to the announcement. These coefficients are not shown and are key to test for an appropriate control group.

Our response: we selected our control *a priori* following the guidance given by https://academic.oup.com/ije/article/47/6/2082/5049576, using the criteria that the control be:

•Independent of intended effect of the SDIL: toiletries do not contain sugar so are not likely to be impacted by the SDIL that leads to the removal of sugar

•Independent of unintended effect of the SDIL: toiletries are not eaten therefore purchased volumes should remain constant as there as it is not likely that consumers will purchase more as an alternative to soft drinks.

•Able to capture background trends: external events such as austerity, Brexit, elections etc. are likely to impact on purchases of both soft drinks and toiletries equally.

•Background trends are likely to be uniform across households: toiletries are purchased by all households regardless of gender, ethnicity, income and age.

We have provided more information justifying this decision on lines 116 to 126 and have included additional text in the first paragraph of the Data Source section.

Due to the significant cost associated with Kantar Worldpanel data we were unable to buy all purchase categories so we are unable to compare a range of potential controls, though we are able to examine the impact of our selected control upon the results by removing it and running uncontrolled models for all drink and confectionery categories. The results of this are presented in Supplementary Tables E and F which show model coefficients for the level and trend following the announcement of the SDIL together with associated 95% confidence intervals. Overall, results were similar in both the controlled and uncontrolled cases though the effect sizes did change. The trend in purchased volume of powdered drinks did reach significant in the uncontrolled cases (though only just) [-0.12 (95% CI -0.23, -0.0002)] and sugar in confectionery did differ significantly following the announcement in the uncontrolled case [-44.4 (95% CI-85.5, -3.3)]. This suggests that the use of toiletries as a control tends towards conservative results. This has been added to the manuscript as sensitivity analysis 3: Uncontrolled interrupted time series analysis.

Also the 56% decrease in volume and sugar content seems very large, given that this was only the announcement, not the implementation of the tax.

Our response: following publication of Scarborough et al., 2020 we are now able to put this in context. There was a 20 percentage point drop in proportion of drinks eligible for levy from Sept 15 to 50 days before implementation from 52% to 32% = 38% relative drop. Whilst this is a large relative reduction within the category of lower levy drinks, these drinks did not make up a large proportion of the overall soft drinks market.

In addition, the paper lacks a contextual framework to hypothesize on the potential changes over the announcement period.

Our response: we have expanded the section in the introduction discussing the context for an analysis in to the announcement period in lines 101 to 115

Introduction

Define in more detail what you mean by series of events (which ones) and complex adaptive systems. I don´t see any application of this in the methods.

Our response: our analysis forms one part of a wider evaluation we are carrying out. The series of events include the announcement, the implementation and the surrounding events that led to the SDIL being announced, the industry and wider economic response. This paper does not examine these complex systems rather provides a piece of evidence examining one part. As our evaluation progresses we will collect more data and plan to synthesise findings to explore wider systemic responses to the SDIL. For clarity, we have amended the paragraph discussing the complex adaptive systems (lines 101 to 115)

Methods

Data- If the data representative of the British population?

Our response: the second paragraph of the results section along with supplementary Table A examine the representation of the panel. As indicated, “The characteristics of included households, after weighting, largely reflected UK households as a whole in 2014-18 (see supplementary Table A).”

Model- Interrupted times series analysis is a method applied to time series. I am not sure that the data is aggregated as time series because the authors are using a panel of households. This is confusing in the methods. Did the authors aggregated the data? At what level and why given the richness of using longitudinal data to explore heterogeneities.

Our response: the data comprise a cross-section of panel households at each weekly time point. We agree that a longitudinal design would provide greater analytical potential; however, the Kantar Worldpanel has a panel of households that changes over time as households leave the panel and can be excluded if they do submit poor quality data. Also the considerable cost associated with these data mean that we do not have access to data on the entire population – we only have those who purchase products from one of the categories. As a result of this a household may remain in the panel but stop purchasing soft drinks. If they purchase alternate drinks this will be captured but if they move to tap water it will not. We are able to provide an average household estimate as we have the total number of households Kantar use to ensure representation but we can’t examine changes in individual households. There are a small number of panel households who record purchases in each week of the study period but these tend to be biased towards older, home owning individuals. We have added a paragraph addressing this in lines 484 to 493

To adjust for seasonality, it is not clear how often temperature is included, monthly, weekly?

Our response: Monthly temperature is included weekly, i.e. there will be four weeks with the same temperature. Lines 222 to 224 have been amended to clarify: “To adjust for seasonality and temperature-related trends in drink consumption the average UK monthly temperature at each weekly time point was included [38].”

Any macroeconomic level variable that is associated with household purchases?

Our response: we are not clear what is referred to by this comment. We did not adjust for inflation. The results presented for an average weekly volume per household are adjusted for the total number of households and this number changes (increases) over the study period.

Control group: why toiletries? It is the only non-food and beverage items included?

Our response: Yes, ‘toiletries’ was the only non-food and beverage category included. We provide a fuller response justifying our use of toiletries as a control category above.

Potential substitutions: I don´t see any justification as for why would confectionary be a substitute for SSB, no literature is cited. What was included in confectionery? How about untaxed beverages? It is very relevant to look at substitutions for no levy beverages.

Our response: we have added further justification to explain that we chose to examine confectionary purchases to examine potential unintended consequences of the SDIL in the case that households reduced their amount of sugar from SSBs but increased purchases of alternate sources of sugar – namely confectionery. We have added references to others who have also speculated that confectionery may be a potential substitute category for SSBs. As stated at lines 145 to 146, confectionery comprised the KWP categories of sugary confectionery and chocolate confectionery combined.

We also examined changes in purchases of the other exempt drinks categories: alcoholic drinks, milk and milk based drinks; no added sugar fruit; and drinks sold as powders to see if households increased purchases of these products while reducing liable drinks and these are presented in tables 2 and 3. It is also worth noting that confectionery was modelled independently of soft drinks, i.e. there was no adjustment in the models for the possibility that purchases may have switched.

The authors should test different inflexion points; right now they are assuming that there was an immediate change after announcement and change in the slope. This could have happened in different times in the post-announcement period.

Our response: we feel that examining other potential inflexion points would be conceptually confusing, prove difficult to interpret and may even result in varied ‘important’ time points for different categories of drink. Whilst it may be the case that the greatest changes were seen at a time point other than the announcement, for example following the launch of reformulated products by a particular manufacturer, we cannot know the driver behind this reformulation. Empirically it appears that the greatest reformulation occurred – at least in the lower tier group – in early 2018 in the months leading up to the implementation [1]. It is likely that this is driven by the announcement and impending implementation rather than some unforeseen event that happened in, say January 2018. We have added a paragraph to this effect to the discussion section (lines 511 to 514).

Results

What are AB or C1 classes?

Our response: these are widely used social grade classifiers in the UK. To clarify in the text we have amended the sentence to “Most panel households did not include children, were in managerial occupations (social grades AB or C1), and earned less than £40,000 per annum” (lines 283 to 285) and amended classes to grades for accuracy.

Table 2 presents two estimations, what are the coefficients in columns 2 and 3 compared to the change at 2 years post-announcement?

Our response: columns 2 and 3 of table 2 refer to the level change and trend changes in the post announcement period relative to the counterfactual based on pre-announcement trends. These are labelled: “Level change (ml/g)” and “Trend change (ml/g per week)” respectively and the text (lines 299 to 302) explains “The level change is the difference between the model estimates and the counterfactual at the first week after the SDIL announcement adjusted for the underlying trends in household purchases captured by the control condition. The trend change is the mean change in the slope of purchases following the announcement”. We have added this explanation as a footnote to Tables 2 and 3 for clarity.

How could there be an increase in sugar in the no levy category? How could reformulation lead to this increase, it makes little sense and the discussion does not provide much inside on these findings.

Our response: As explained in the introduction and restated throughout the manuscript, the no levy category includes drinks with <5g of sugar per 100ml, it is not a zero-sugar category. Sweetened soft drinks are sweetened with sugar, artificial sweeteners or a combination of both. Following the announcement manufacturers were presented with thresholds for the amount of sugar (≥5g-8g and ≥8g of sugar per 100ml) in taxable drinks. Substantial reformulation has been observed such that a much higher proportion of soft drinks now contain <5g of sugar per 100ml than before the levy was announced [1]. However, many of these reformulated drinks do not contain zero grams of sugar – reformulation appears to have been achieved by replacement of some, but not all, sugar with artificial sweeteners. Indeed, a new peak in the distribution of sugar concentration across drinks is observed at just less than 5g sugar per 100ml.

This point is already noted in the results section at lines 331 to 335. We have added further text to the discussion to clarify this point on lines 443 to 448: “We hypothesise that these results reflect reformulation of many drinks to just below the maximum sugar content for the no levy tier (<5g of sugar per 100ml) and that this led to an overall increase in the average sugar content of drinks in this category. Prior to the levy announcement, this category was largely populated with zero sugar drinks, but after implementation a substantial new group of drinks with between 4.5g and 4.9g of sugar was seen [26].”

**Reviewer #2:**

This was a well-conducted and reported time series analysis used for policy evaluation. The methods are robust and the author explained their methods and approach clearly, which can be sometimes particularly challenging the reporting of ITS studies for a more general medical journal such as PLOS Medicine. The study itself is timely and novel and should generate some substantial impact as many policy-makers are waiting for this type of analysis to be conducted evaluating the UK SDIL. The panel dataset used to examine this was from a large number of household (30,000 members) and covered over 27 million purchases. Only a small amount of imputed data was necessary (0.5%) - suggesting the data was mostly complete. This paper should warrant publication barring a few minor issues to addressed first I've detailed below.

Our response: many thanks for this positive evaluation of our paper. We are particularly pleased that the reviewer felt we had communicated this, often difficult to explain, method well.

1) Abstract - primary outcome: give the unit of measurement for both volume and amount of sugar in these outcomes.

Our response: carried out as requested

2) Abstract - results: I it would make more sense to have a "-" sign in front the absolute reduction of volume and amount of sugar figures, to ensure consistency with the reported 95% CI

Our response: carried out as requested

3) Introduction (and related methods): Selection of toiletries as the control - I don't disagree with the authors rationale on why this was selected as the control but in terms of the hypothesis that toiletries were sensitivity to disposable income? However, could the authors elaborate if they looked in their data whether this was in fact sensitive to disposable income? A simple exploration of the relationship between income and toiletry purchasing could confirm this as this looks to be one the key assumptions on control selection.

Our response: we provide a detailed response further justifying our selection of toiletries as the control category above. Sensitivity to disposable income was not, in fact, a key criterion when selecting the control category and we have removed reference to this.

4) Main analysis methods - Was there any testing or investigation of stationarity in both the experimental and counterfactual models? The design of having a counterfactual trend for toiletries for comparison partially controls for this issue, but assumes both products have similar levels of stationarity.

Our response: stationarity in time series trends was examined. We have included a sentence (lines 217 to 219) to this effect “No evidence of stationarity in each time series of volume and sugar was found using augmented Dickey-Fuller tests (both without and with trend).”

5) Main analysis methods - Did the authors consider any changes in household size of period of time their analysis period is over four year period - consumptions patterns change or behavioural patterns may changes due to households over time due to external influences (i.e. children)

Our response: the panel is refreshed as households leave and new ones join. This is done according to population-based quotas to ensure the panel does not become biased towards a particular socio-demographic group. The average number of people per household remained constant over the 4 year period at 2.69 people per household on average in the period before the announcement and 2.69 people per household on average in the period following the announcement. We have amended lines 281 to 284 to state that household size did not change: “The characteristics of included households, **including household size**, remained consistent over the study period.”

6) Main analysis method - The authors considered the effects of period based purchasing (i.e. Christmas, Easter) using dummy indicator variables but I was wondering if the models were seasonally adjusted or included a seasonal term

Our response: model specification is detailed in lines 199 to 237. As stated, we included a continuous variable indicating the average monthly temperature to capture variations in household purchases during summer and other seasonal trends in purchasing. We have amended lines 222 to 224 to emphasis this “To adjust for **seasonality and** temperature-related trends in drink consumption the average UK monthly temperature at each weekly time point was included [38]”

7) Generating 95% CI with multivariate delta method: Explain the method is used to generate estimations of sampling variance (hence able to determine 95% CIs)

Our response: we have expanded the section (lines 230 to 240) to explain that “Absolute and relative differences between observed post-announcement purchasing and the counterfactual scenario (assuming pre-announcement trends continued post-announcement) at 105 weeks (‘2 years’) post-announcement are presented. Ninety-five percent confidence intervals for absolute differences are given by $(\hat{Y}_{t105w}-\hat{Y}_{t105}) \pm1.96*\sqrt{VAR(\hat{Y}_{t105w}- \hat{Y}_{t105})}$ Where $\hat{Y}_{t105w}$ is the estimated purchased volume or amount of sugar given the SDIL announcement took place after 105 weeks and $\hat{Y}_{t105}$ is the counterfactual at 105 weeks post announcement, and VAR refers to the variance [39]. To calculate 95% confidence intervals for the relative change by dividing the absolute difference by $\hat{Y}_{t105}$ would inflate the amount of variance giving incorrect values. Therefore 95% confidence intervals for the relative difference were obtained following the multivariate delta method which uses Taylor series expansion to estimate the relative variance [39].”

8) Sensitivity analyses - As the authors can appreciate, public health policy has differential treatment effects due to socioeconomics and levels of education. I noticed in the descriptive tables, there were figures on household income and social class. I was expecting to see a sensitivity analysis in this paper stratifying the effects of SIL by SES. What the key questions would have been very desirable to see if what effect SIL had between these stratums - and in fact would further enhance the overall findings.

Our response: we agree with the comment that it is important to explore the impact of the SDIL across socio-demographic groups and specified this in our protocol*. However we feel that the current manuscript examining the impact of the announcement across the two levied drinks categories, the eight SDIL exempt categories and confectionery is already very full and that we would prefer to devote a further paper to concentrating on differential effects by SES together with the impact on households with children. This is already stated in our section on “Changes to protocol” (lines 266 to 275).

*https://njl-admin.nihr.ac.uk/document/download/2010886

9) Again for consistency in results section - I would suggest having "-" in front when presenting reductions which would correspond to the reported 95% CIs.

Our response: carried out as requested.

**Reviewer #3:**

There has been much speculation about the time course of any changes in SSB consumption associated with the announcement and subsequent implementation of the SDIL. This analysis is thus welcome.

Our response: We are pleased that the reviewer considers the analyses presented to be valuable.

ABSTRACT

The manuscript-based abstract correctly commences the Results section with the key finding.

"There was no evidence that volume of, or amount of sugar in, purchases of all drinks combined was different from the counterfactual."

This sentence appears to have been mistakenly moved to the end of the results in the web-based abstract. That should be corrected.

Our response: an updated abstract has been provided and we have ensured this is consistent in both the manuscript and on the web system. As all drinks was not our primary outcome, we have chosen to report this last rather than first.

DISCUSSION

Para 1 is potentially confusing. I suggest that before embroiling the reader in the detail, the second sentence might usefully start with the key overall message. Something along the lines of:

"We theorised that the announcement of the SDIL might lead to anticipatory changes, both by industry (as intended by government) and by consumers (via changes in consumer awareness, attitudes or beliefs).

When all drinks were combined, we found no significant change in the volume of, or amount sugar in, purchased drinks."

Our response: we have added the sentence to the discussion as suggested

The Concluding para ends:

"Overall there was no change in total volume of, or sugar in, household purchases of all soft drinks combined indicating that further action, including implementation of the SDIL, is required to achieve public health impact. Future work should determine the impacts of the implementation of the SDIL."

This is slightly disappointing. It would be preferable to have one paper showing all the results together, to also see the changes post SDIL implementation.

Our response: we respectively disagree. We are certainly working on post-implementation analyses but this will take time. Given the policy relevance and fast moving nature of policy internationally in this space, we think there is value in sharing these early results with researchers and the policy community which, alongside Scarborough et al., 2020 and Bandy et al., 2020, indicate that there is substantial movement ahead of implementation. This should be useful with respect to political discussions about rescinding sugar taxes (which has happened in, for example, Catalonia, Denmark and Cook County, Illinois, and is not therefore merely a theoretical concern). It should also help countries implementing similar taxes to understand the timeline they might expect for effects to occur (and hence when evaluations should take place and when any impact on health outcomes may be observed). It also reflects our theoretical framework in which we think reformulation and signalling might happen early and helps us build a rich picture, in due course, of what happened, how and why. The alternative is not to publish anything until all the evidence is available - which is obviously also not ideal. In this paper we have tried to steer a useful course between these two poles. We note that the other reviewers are supportive of publication at this time. As noted above, we have added additional text to the introduction to clarify the conceptual framework of our evaluation and the value of these early results as contributing to an evolving whole (lines 101 to 115).

References

[1] Scarborough et al., Impact of the announcement and implementation of the UK Soft Drinks Industry Levy on sugar content, price, product size and number of available soft drinks in the UK, 2015-19: A controlled interrupted time series analysis, <https://journals.plos.org/plosmedicine/article?id=10.1371/journal.pmed.1003025>

[2] Bandy et al., Reductions in sugar sales from soft drinks in the UK from 2015 to 2018, <https://bmcmedicine.biomedcentral.com/articles/10.1186/s12916-019-1477-4>
