# Supplementary material for: Anticipatory changes in British household purchases of soft drinks associated with the announcement of the Soft Drinks Industry Levy: A controlled interrupted time series analysis
Source: PLoS Med. 2020 Nov 12;17(11):e1003269. doi: 10.1371/journal.pmed.1003269 (PMC7660521; doi:10.1371/journal.pmed.1003269)
Supplement: Attachment — Submitted filename: Response to comments.docx [file pmed.1003269.s011.docx]

Requests from Editors:
Title- please remove [ISRCTN18042742] from the title
Response – done

Competing interests- could you please add a sentence to note that Jean Adams is an Academic Editor for PLOS Medicine

Response – done

References- please adapt to Vancouver style

Response – done

Abstract- limitations should be made more explicit. For instance instead of "Only purchases brought into the home were included" you may say "out of home purchases could not be accounted for" or similar.
Response – amended as suggested

Page 5- please add a subheading “Author Summary” and remove the gray background here please
Response – done

Author summary in the section “what do these findings mean” please can you tone down to remove causal language? For instance “Households started changing what they purchased in the two year period between announcement and implementation of the SDIL either because households were aware of the tax and purchased different drinks or more likely because manufacturers removed the sugar in these drinks”
Response – amended as suggested

Please provide p values where needed, especially where you mention “significance” for example page 18 and Table 2,3. These should be exact p values unless p<0.001

Response - done

Comments from Reviewers:

Reviewer #2: I have re-reviewed the manuscript and found the authors addressed most of the reviewer comments adequately.

I had one minor issue I was mulling over about in the additional supplemental analysis Tables E and F. The results are somewhat similar, with expected changes in effect sizes. The trends did reach statistical significance for purchase volume of powdered drinks and sugar in confectionary in the uncontrolled analysis. I agree that this would mean the use of controls was conservative choice but is there any type 2 error risk here with the choice of the control? The choice for toiletries as the control was largely pragmatic which I understand but I think a limitation is that the researcher could not access other household products to investigate this issue further. This should probably just be mentioned as a limitation of the analysis.

Response – there has been some minor updates to the analysis. They were implemented in the uncontrolled analysis but not in the controlled case hence the larger than expected differences. When the updated controlled analysis is compared to the uncontrolled the differences are much less pronounced. Nevertheless, we have added a comment relating to the a priori selection of toiletries in the text as suggested.
