# Supplementary material for: Correction: Anticipatory changes in British household purchases of soft drinks associated with the announcement of the Soft Drinks Industry Levy: A controlled interrupted time series analysis
Source: PLoS Med. 2023 Mar 13;20(3):e1004201. doi: 10.1371/journal.pmed.1004201 (PMC10010746; doi:10.1371/journal.pmed.1004201)
Supplement: S1 File — (PDF) [file pmed.1004201.s001.pdf]

PLOS MEDICINE

RESEARCH ARTICLE

Anticipatory changes in British household purchases of soft drinks associated with the announcement of the Soft Drinks Industry Levy: A controlled interrupted time series analysis

David Pell[1], Tarra L. Penney[1,2], Oliver Mytton[1], Adam Briggs[3,4], Steven Cummins[5], Mike Rayner[6], Harry Rutter[7], Peter Scarborough[3,6], Stephen J. Sharp[1], Richard D. Smith[8], Martin White[1], Jean Adams[1]*

1 Centre for Diet and Activity Research, MRC Epidemiology Unit, University of Cambridge School of Clinical Medicine, Institute of Metabolic Science, Cambridge, United Kingdom, **2** Faculty of Health, School of Kinesiology & Health Science, York University, Toronto, Canada, **3** Centre on Population Approaches for Non-Communicable Disease Prevention, Nuffield Department of Population Health, University of Oxford, Headington, Oxford, United Kingdom, **4** Warwick Medical School, The University of Warwick, Division of Health Sciences, Coventry, United Kingdom, **5** Population Health Innovation Lab, Department of Public Health, Environment and Society, London School of Hygiene and Tropical Medicine, London, United Kingdom, **6** National Institute of Health Research Oxford Biomedical Research Centre, Oxford University Hospitals NHS Foundation Trust, Headington, Oxford, United Kingdom, **7** Department of Social and Policy Sciences, University of Bath, Claverton Down, Bath, United Kingdom, **8** College of Medicine and Health, University of Exeter, Medical School Building, St Luke's Campus, Exeter, United Kingdom

* jma79@medschl.cam.ac.uk

OPEN ACCESS

Citation: Pell D, Penney TL, Mytton O, Briggs A, Cummins S, Rayner M, et al. (2020) Anticipatory changes in British household purchases of soft drinks associated with the announcement of the Soft Drinks Industry Levy: A controlled interrupted time series analysis. PLoS Med 17(11): e1003269. https://doi.org/10.1371/journal.pmed.1003269

Academic Editor: Barry M. Popkin, Carolina Population Center, UNITED STATES

Received: September 23, 2019

Accepted: September 18, 2020

Published: November 12, 2020

Peer Review History: PLOS recognizes the benefits of transparency in the peer review process; therefore, we enable the publication of all of the content of peer review and author responses alongside final, published articles. The editorial history of this article is available here: https://doi.org/10.1371/journal.pmed.1003269

Copyright: © 2020 Pell et al. This is an open access article distributed under the terms of the Creative Commons Attribution License, which permits unrestricted use, distribution, and reproduction in any medium, provided the original author and source are credited.

Data Availability Statement: The statistical code for the analyses are available from https://github.com/MRC-Epid/SDILEvaluation. Kantar Worldpanel

Abstract

Background

Sugar-sweetened beverage (SSB) consumption is positively associated with obesity, type 2 diabetes, and cardiovascular disease. The World Health Organization recommends that member states implement effective taxes on SSBs to reduce consumption. The United Kingdom Soft Drinks Industry Levy (SDIL) is a two-tiered tax, announced in March 2016 and implemented in April 2018. Drinks with ≥8 g of sugar per 100 ml (higher levy tier) are taxed at £0.24 per litre, drinks with ≥5 to <8 g of sugar per 100 ml (lower levy tier) are taxed at £0.18 per litre, and drinks with <5 g sugar per 100 ml (no levy) are not taxed. Milk-based drinks, pure fruit juices, drinks sold as powder, and drinks with >1.2% alcohol by volume are exempt. We aimed to determine if the announcement of the SDIL was associated with anticipatory changes in purchases of soft drinks prior to implementation of the SDIL in April 2018. We explored differences in the volume of and amount of sugar in household purchases of drinks in each levy tier at 2 years post announcement.

Methods and findings

We used controlled interrupted time series to compare observed changes associated with the announcement of the SDIL to the counterfactual scenario of no announcement. We

data are not publicly available but can be purchased from Kantar Worldpanel (http://www.kantarworldpanel.com). The authors are not legally permitted to share the data used for this study, but interested parties may contact Kantar WorldPanel representative Sean Cannon to inquire about accessing this proprietary data (Sean.Cannon@kantar.com).

Funding: DP, TLP, OM, MW and JA were funded by the Centre for Diet and Activity Research (CEDAR) (https://www.cedar.iph.cam.ac.uk/), a UKCRC Public Health Research Centre of Excellence. Funding from the British Heart Foundation, Cancer Research UK, the Economic and Social Research Council, the Medical Research Council, the National Institute for Health Research, and the Wellcome Trust, under the auspices of the UK Clinical Research Collaboration, is gratefully acknowledged. This work was supported by the Medical Research Council MC_UU_12015/6 and by the NIHR Public Health Research programme (project No 16/130/01) (https://www.nihr.ac.uk/explore-nihr/funding-programmes/public-health-research.htm). The funders had no role in study design, data collection and analysis, decision to publish, or preparation of the manuscript.

Competing interests: I have read the journal's policy and the authors of this manuscript have the following competing interests: AB is a co-applicant on the National Institute for Health Research grant number 16/130/01, evaluating the impact of the UK Soft Drink Industry Levy, and is a past member of the UK Health Forum and current member of the Faculty of Public Health. Both of these organisations have a position statement supporting a soft drink tax. MR is Chair of Sustain: The alliance for better food and farming, a UK-based NGO. Sustain has advocated for the introduction and development of the SDIL. MW is director of the NIHR Public Health Research Funding programme; OM is currently on secondment at the UK Department of Health and Social Care and previously worked with Public Health England. JA is an academic editor for *PLOS Medicine*. Other than this, there was no support from any organisation for the submitted work other than that described above; no financial relationships with any organizations that might have an interest in the submitted work in the previous 3 years; and no other relationships or activities that could appear to have influenced the submitted work.

Abbreviations: CI, confidence interval; ITS, interrupted time series; KWP, Kantar Worldpanel; NHS, National Health Service; SDIL, Soft Drinks Industry Levy; SSB, sugar-sweetened beverage;

used data from Kantar Worldpanel, a commercial household purchasing panel with approximately 30,000 British members that includes linked nutritional data on purchases. We conducted separate analyses for drinks liable for the SDIL in the higher, lower, and no-levy tiers controlling with household purchase volumes of toiletries. At 2 years post announcement, there was no difference in volume of or sugar from purchases of higher-levy-tier drinks compared to the counterfactual of no announcement. In contrast, a reversal of the existing upward trend in volume (ml) of and amount of sugar (g) in purchases of lower-levy-tier drinks was seen. These changes led to a −96.1 ml (95% confidence interval [CI] −144.2 to −48.0) reduction in volume and −6.4 g (95% CI −9.8 to −3.1) reduction in sugar purchased in these drinks per household per week. There was a reversal of the existing downward trend in the amount of sugar in household purchases of the no-levy drinks but no change in volume purchased. At 2 years post announcement, these changes led to a 6.1 g (95% CI 3.9–8.2) increase in sugar purchased in these drinks per household per week. There was no evidence that volume of or amount of sugar in purchases of all drinks combined was different from the counterfactual. This is an observational study, and changes other than the SDIL may have been responsible for the results reported. Purchases consumed outside of the home were not accounted for.

Conclusions

The announcement of the UK SDIL was associated with reductions in volume and sugar purchased in lower-levy-tier drinks before implementation. These were offset by increases in sugar purchased from no-levy drinks. These findings may reflect reformulation of drinks from the lower levy to no-levy tier with removal of some but not all sugar, alongside changes in consumer attitudes and beliefs.

Trial registration

ISRCTN Registry ISRCTN18042742.

Author summary

Why was this study done?

- Consumption of sugar-sweetened beverages (SSBs) is associated with dental caries, obesity, type 2 diabetes, and cardiovascular disease.
- In March 2016, the UK government announced a tax on SSB manufacturers and producers, called the Soft Drinks Industry Levy (SDIL).
- The SDIL has a higher tier of £0.24 per litre on drinks containing $\geq$8 g of sugar per 100 ml and a low tier of £0.18 per litre on drinks containing 5 to $<$8 g of sugar per 100 ml.
- The SDIL was announced 2 years before it was implemented to allow manufacturers time to reformulate products and so may have led to changes in purchases of SSBs even before it was implemented.

STROBE, Strengthening the Reporting of Observational Studies in Epidemiology.

What did the researchers do and find?

- We analysed purchases of drinks in the two levy tiers and other non-taxed drinks categories from 2014 to 2018 covering 2 years before the SDIL was announced and the 2 years after it was announced before it was implemented.
- We accounted for the increase in purchases that occurred in the weeks leading up to Christmas and Easter, the fall in purchases in the weeks after Christmas, and temperature, as purchases tend to increase during warmer months.
- We found that the purchased volume of and the amount of sugar in low-tier drinks reduced but that the purchased amount of sugar in the no-levy category (containing drinks with <5 g of sugar per 100 ml) increased; this may be because drinks from the taxed categories had enough sugar removed to be exempt from the tax and therefore moved in to the no-levy category.
- The announcement of the SDIL was not associated with changes in household purchases of higher-tier drinks; this was against a backdrop of a strong existing downward trend in purchases of these drinks.

What do these findings mean?

- Taxes on SSBs are becoming more common throughout the world, but they vary in design. Households started changing what they purchased in the 2-year period between announcement and implementation of the SDIL because either they were aware of the tax and purchased different drinks or, more likely, manufacturers removed the sugar in these drinks.
- Overall, the announcement of the SDIL was associated with no change in the total amount of sugar purchased in drinks; further action, including implementation of the SDIL, may be required to achieve public health benefit.

Introduction

Sugar-sweetened beverage (SSB) consumption is positively associated with dental caries, total energy intake, obesity, type 2 diabetes, and cardiovascular disease [1–3]. Each additional daily serving of SSBs consumed on a regular basis is associated with an 18% increased risk of type 2 diabetes and a 17% increased risk of coronary heart disease [2,3]. The economic burden of this is significant. Obesity cost the UK economy around £27 billion in 2015 [4], with direct costs to the UK National Health Service (NHS) of over £5 billion [5].

The World Health Organization recommends that member states implement effective taxes on SSBs to reduce consumption [6,7]. A number of national and regional governments including Mexico, France, and multiple United States cities have introduced SSB taxes [8–14]. Although the longer-term impacts of these taxes upon obesity rates are yet to be observed, short- and medium-term investigations have reported a drop in both SSB purchasing and consumption [10,12,15–17]. Many of these taxes generate an associated increase in the price of SSBs [9,18,19], which may be responsible for impacts on purchasing and consumption.

However, there may also be other mechanisms of effect, including signalling of the health risks associated with SSBs, changes in social norms, reduced portion sizes, and reformulation of drinks [20]. To date, these alternative mechanisms have received less research attention.

On 16 March 2016, the UK government announced the Soft Drinks Industry Levy (SDIL). The SDIL was the first SSB tax explicitly designed to incentivise a reduction in the amount of sugar in SSBs through reformulation [21]. This is reflected in the two levy tiers: £0.24 per litre for drinks containing ≥8 g total sugar per 100 ml and £0.18 per litre for drinks containing ≥5 g and <8 g total sugar per 100 ml. Drinks containing <5 g total sugar per 100 ml are not taxed. Drinks containing at least 75% milk or milk alternatives, low- and no-alcohol drinks marketed as direct replacements for alcoholic drinks with <1.2% alcohol by volume, no-added-sugar fruit juice, drinks sold as powders, alcoholic drinks with >1.2% alcohol by volume, infant formula, and drinks for special medical purposes are exempt from the SDIL irrespective of sugar content. Products from small manufacturers and producers with annual sales of <1 million litres of liable drinks are also exempt [22]. A number of other countries, including Ireland [23] and South Africa [24], have recently introduced similar taxes based on sugar concentration.

The announcement of the SDIL included a stated implementation date of April 2018 [15], giving manufacturers 2 years to adapt (e.g., reformulate their products or introduce new ones) prior to implementation. The announcement received extensive media coverage [25] and, together with discussion surrounding the potential harms of SSBs, may have itself impacted purchasing and consumption via changes in attitudes and beliefs. Furthermore, manufacturers had begun to introduce reformulated and new, lower-sugar products during the 2-year adaptation period. The availability of SDIL-liable soft drinks on supermarket shelves fell 19.5% almost 2 years after the announcement of the SDIL [26]. Although it has been reported that sales of levy-eligible soft drinks fell by 50% from 2015 to 2018, leading to an overall reduction in the amount of sugar in purchased soft drinks of 30% [27], this before-after study was not able to distinguish the impact of the SDIL from other trends in soft drinks purchases.

In line with recent developments in the public health literature [28], our evaluation theorised the SDIL as a series of events (specifically the announcement and implementation of the levy and related responses from relevant actors) in a complex adaptive system and planned analyses to evaluate the impact of each event [29]. In this framing, the SDIL announcement forms an important early phase of the intervention that is related to, but distinct from, the implementation. The 2 years between announcement and implementation was intended to give soft drinks manufacturers and producers time to respond to the SDIL under the assumption that it would be implemented. During this period, changes in the availability of soft drinks occurred [26], which appear to have specifically been in anticipation of the implementation. It is important to explore the impact of this preparatory stage because, were the levy to be repealed, these anticipatory responses may not be reversed. By studying the impact of the SDIL on household purchases of soft drinks following the announcement along with postimplementation changes and other work examining the impact on health outcomes, the wider food and drinks industry and economy, and integrating findings in our overall SDIL evaluation [30], we aim to build up a comprehensive picture of the impacts of the SDIL and the mechanisms through which those were achieved [30].

In this paper, we aimed to determine if the announcement of the SDIL was associated with anticipatory changes in purchases of soft drinks prior to implementation of the SDIL in April 2018 [28]. We have explored differences in the volume of or amount of sugar in household purchases of drinks in each levy tier, exempt drinks categories, and confectionery at 2 years post announcement. We used controlled interrupted time series (ITS) methods with toiletries included as a control category to take account of underlying trends in household purchasing, which we hypothesised to be unaffected by the SDIL. This allows existing purchasing trends to

be taken into account when comparing pre- to postannouncement data, and prediction of longer-term changes in purchasing, compared to the counterfactual scenario. We compared observed changes associated with the announcement of the SDIL to the counterfactual scenario in which the announcement did not take place. The protocol was published [29] and the study was registered (ISRCTN18042742) [30].

Methods

National-level policy interventions such as the SDIL are not amenable to evaluation using randomised controlled trials. Controlled ITS analysis offers a robust observational method that allows the impact of the announcement to be investigated by examining both immediate changes in purchases and trends in these over time in comparison to counterfactual scenarios [31]. The counterfactual is the trend that would have occurred if the SDIL was not announced and is estimated by extrapolating the preannouncement trend. A controlled design that uses a product category likely to be unaffected by the announcement of the SDIL (i.e., toiletries) takes account of underlying trends in overall household purchasing [32,33]. In this study, our primary outcomes were differences in the volume of and amount of sugar in purchases of drinks in each levy tier and exempt categories per household per week compared to the counterfactual scenario of no announcement, at 2 years post announcement. To assess whether households would consciously or subconsciously maintain their sugar intake by switching from SSBs to alternate high-sugar products [34,35], we also studied trends in purchased total pack weight and sugar content of confectionery.

This study is reported as per the Strengthening the Reporting of Observational Studies in Epidemiology (STROBE) guideline (S1 Checklist).

Data source

We used routinely available data from a commercial household purchasing panel, with approximately 30,000 British members, that includes linked nutritional data on food and drink purchases (Kantar Worldpanel [KWP] aggregated to the weekly level). This allows purchases of SSBs to be examined in detail over time and compares favourably to other measures of food purchases [36]. Household purchasing of all drinks (including alcoholic drinks), sugar confectionery and chocolate confectionery (referred to collectively here as ‘confectionery’), and shampoo, conditioner, and liquid soap (referred to collectively here as ‘toiletries’) recorded by KWP households between 3 March 2014 and 25 March 2018 were included. We selected shampoo, conditioner, and liquid soap from the wider category of all health and beauty products on the basis that they were not seasonally dependent (such as for sun cream), not likely to be impacted by changes in sugar consumption (such as toothpaste), not likely to be impacted by households composition (such as gender-biased products like make-up), and were sold by volume (rather than units unlike, for example, soap bars) and that purchased volumes were of a similar magnitude to drink purchases. Panel households record information by scanning the barcode of all purchases brought into the home. Panel members receive points, exchangeable for gift vouchers worth approximately £100 per year, as an incentive for taking part, and report household demographic information every 4 weeks.

Purchase data are sent electronically from participating households to KWP each week and linked to nutritional compositional data (including sugar content) collected on a rolling basis by KWP field-workers, covering all products every 6 months. Thus, nutritional data associated with each product in KWP change over time in response to changes on product labels. Composition data for new products are collected when 20 purchases have been recorded within a 3-month period. KWP field-workers visit supermarkets and photograph nutritional

information panels identified by their barcode. Photographs are transcribed and linked via barcodes to purchasing records.

When a product cannot be found in any supermarket, nutritional information is substituted from elsewhere. Ideally, data from a different sized product in the same brand are used (e.g., nutritional information from a 500-ml bottle for a 330-ml can). If no alternative within the same brand exists, the mean nutritional data of all similar products are imputed (e.g., all cola drinks). When a product included a mix of imputed and observed sugar content values over the study period, we replaced imputed values by the last previously observed valued. As nutritional information panels are not generally displayed on alcoholic drinks in the UK, we did not study sugar in purchases of alcoholic drinks. Products in other categories that contained only imputed sugar values were excluded. This included all products in the skimmed milk category. We checked the validity of the remaining nutritional data and found that it was highly correlated with contemporaneous nutritional data on supermarket websites (see S6 Text) and that the imputed products were spread evenly across drinks categories.

Households that record five or fewer purchases per week are excluded by KWP along with households whose adjusted weekly spending does not meet an undisclosed proprietary minimum value. KWP applies weights to purchases to adjust for households excluded because of minimum purchase or spending thresholds and to maintain the representativeness of the panel. These weights were used in all analyses and ensure that the panel, and all purchases within it, are considered representative of all British households and purchases. In particular, our data represent mean purchases per household per week across all British households including nonpurchasing households, rather than across all British households that purchased a particular product or group of products.

Drink categories

Drinks liable for the SDIL were classified into three categories: drinks containing ≥8 g total sugar per 100 ml (higher levy tier); drinks containing ≥5 g to <8 g total sugar per 100 ml (lower levy tier); and drinks containing <5 g total sugar per 100 ml (no levy). Nonexempt drinks containing <5 g total sugar per 100 ml were subcategorised into flavoured drinks containing >0 g to <5 g total sugar per 100 ml, flavoured drinks containing 0 g of sugar per 100 ml, and bottled water. Exempt drinks were categorised as alcoholic drinks containing >1.2% alcohol by volume and drinks with less alcohol by volume that are marketed as direct replacements for alcoholic drinks (collectively termed 'alcoholic drinks'); milk and milk-based drinks containing at least 75% milk or milk alternatives (e.g., soy and almond drinks); no-added-sugar fruit juice; and drinks sold as powders (e.g., teas, coffees, and hot chocolate). The SDIL includes further exemptions for infant formulas and foods for special medical purposes that were not examined [21].

Analysis

Main analysis. We conducted separate controlled ITS analyses for each drink category and confectionery controlling for household purchase volumes of toiletries. We used 212 weekly time points from 3 March 2014 to 25 March 2018 giving 107 pre- and 105 postannouncement weeks. The model is specified as follows:

$$Y_t = \beta_0 + \beta_1 T_t + \beta_2 X_t + \beta_3 X_t T_t + \beta_4 Z + \beta_5 Z T_t + \beta_6 Z X_t + \beta_7 Z X_t T_t + e_t$$

where Y_t is the outcome (average weekly household purchases) at time t, β_0 to β_3 are the coefficients for the control group, and β_4 to β_7 represent the intervention group (drink category or confectionery). T_t represents the number of weeks since the first time point, a dummy variable

is given by X_t where 0 indicates the period prior to the SDIL announcement and 1 indicates the period after the announcement, and X_tT_t is the interaction of the announcement and the time since the start of the study allowing the trend following the announcement to be modelled. The control group (toiletries) is included through Z, a dummy variable indicating drink or confectionery category or control category; β_4 gives the difference between the treatment and counterfactual before the announcement. The difference in the slope between the treatment and control groups before the announcement is given by β_5, and the difference in the level in the week immediately after the announcement is given by $\beta_{6;}$ β_7 gives the difference between the slopes in the treatment and control groups after the announcement, and e_t is the variability at time t not explained by the model [37]. No evidence of stationarity in each time series of volume and sugar was found using augmented Dickey-Fuller tests (both without and with trend). Dummy indicator variables determined to be statistically significant ($p < 0.05$) were included as appropriate representing: the increase in purchases seen throughout December in the weeks before Christmas; the fall in purchases in the weeks immediately after Christmas; and the increase in confectionery purchases seen at Easter. To adjust for seasonality and temperature-related trends in drink consumption, the average UK monthly temperature at each weekly time point was included [38]. Quadratic functions of trend TX_t were included where they improved model fit—assessed using likelihood ratio tests. Autocorrelation between preceding time points was examined using Durbin-Watson tests and autocorrelation and partial-autocorrelation plots. An appropriate autocorrelation structure was determined and then compared to alternative models using likelihood ratio tests. Visual inspection of the data suggested no additional benefit would be gained from including polynomial terms.

Absolute and relative differences between observed postannouncement purchasing and the counterfactual scenario (assuming preannouncement trends continued post announcement) at 105 weeks ('2 years') post announcement are presented. Ninety-five percent confidence intervals (CIs) for absolute differences are given by:

$$(\hat{Y}_{t105w} - \hat{Y}_{t105}) \pm 1.96 * \sqrt{VAR(\hat{Y}_{t105w} - \hat{Y}_{t105})}$$

where $\hat{Y}_{t105w}$ is the estimated purchased volume or amount of sugar given the SDIL announcement took place after 105 weeks and $\hat{Y}_{t105}$ is the counterfactual at 105 weeks post announcement, and VAR refers to the variance [39]. To calculate 95% CIs for the relative change by dividing the absolute difference by $\hat{Y}_{t105}$ would inflate the amount of variance giving incorrect values. Therefore, 95% CIs for the relative difference were obtained following the multivariate delta method, which uses Taylor series expansion to estimate the relative variance [39].

Sensitivity analysis 1: Exemptions for small manufactures and producers. Soft drinks manufacturers and producers with annual sales of <1 million litres of liable drinks are excluded from the levy. Relevant manufacturers are required to self-identify to Her Majesty's Revenue and Customs via their tax returns. A list of exempt manufacturers was not available to us; therefore, in the main analysis, products from all manufacturers were included. To estimate whether results were impacted by excluding smaller manufacturers, we estimated annual sales per manufacturer by summing purchases of liable drinks by manufacturer within each year. Thus, in this sensitivity analysis, we repeated the analysis described above firstly excluding liable products from manufacturers and producers with less than an average of 1 million litres per year in our dataset. In addition, as the KWP data we used only capture purchases brought in to the home, they underestimate total sales; therefore, we performed a further set of analyses excluding manufacturers with less than a more conservative average of 0.5 million litres per year.

Sensitivity analysis 2: Combining drinks categories. The SDIL does not apply to products such as fruit juices and milk-based drinks that may contain comparable amounts of sugar to SDIL-liable products. To examine the extent to which the SDIL impacted upon the purchased volume and amount of sugar in all soft drinks, regardless of their SDIL liability, we also examined purchases of all nonalcoholic drinks combined. Controlled ITS analysis was carried out as above using all drinks categorised using the SDIL tier thresholds, as well as all drinks combined.

Sensitivity analysis 3: Uncontrolled ITS analysis. A priori, we selected toiletries as a suitable control category for the reasons described above. It is possible, however, that a more appropriate control exists or that 'no category' is an appropriate control. We were not able to examine alternative controls, but we are able to examine the impact of the selected control on the results we present. To this end, we replicated the main analyses for drink and confectionery categories as described above with no control.

Changes to protocol

A number of changes to the published protocol were made. First, rather than use data from 2 full years pre- to 2 full years post announcement, we included data from 107 weeks pre- to 105 weeks post announcement, reflecting a small amount of additional data made available to us by KWP. Second, we did not include data on out-of-home purchases, as indicated in the protocol. Although KWP does have a recently established panel capturing out-of-home purchases, data are only available on a subset of households and only from June 2015, and we did not feel this would provide a robust preintervention period. Third, we analysed purchasing at the weekly rather than 4-weekly level, reflecting an advantageous change in the data that KWP made available to us. Further analyses specified in the protocol [29] will be presented in future papers.

Results

Of approximately 27 million purchases in the drinks, confectionery, and toiletries categories over 212 weeks, 7% were for nonalcoholic drinks with only imputed sugar values and were excluded from the analyses. A further 0.5% contained a mix of imputed and observed values and were retained with last observed values carried forward.

An average of 22,265 households reported purchases in included categories each week. The characteristics of included households, including household size, remained consistent over the study period. Most panel households did not include children, were in managerial occupations (social grades AB or C1), and earned less than £40,000 per annum. Less than a third of chief income earners in included households had a degree-level education. The characteristics of included households, after weighting, largely reflected UK households as a whole in 2014–2018 (see S1 Text) [40–43].

Table 1 presents the unadjusted mean purchases of drinks in each category, confectionery, and toiletries per household per week pre- and post announcement. Overall, mean volume of levy-liable drinks purchased per household per week was 284 ml lower in the post- versus pre-announcement period, with a corresponding 30.4 g reduction in the amount of sugar purchased per week. This was primarily attributable to a reduction in purchasing of drinks in the higher levy tier.

Summaries of the controlled ITS models are shown in Tables 2 and 3. These tables document level and trend changes in the volume of and amount of sugar in purchases per household per week and absolute and relative differences 2 years post announcement, compared to the counterfactual. The level change is the difference between the model estimates and the

Table 1. Unadjusted mean (sd) volume of and amount of sugar in purchased drinks and confectionery per household per week pre- and post announcement of the SDIL, March 2014 to March 2018.

	Mean (sd) volume (ml/g)		Mean (sd) amount of sugar (g)	
**Category**	**Pre-SDIL announcement**	**Post SDIL announcement**	**Pre-SDIL announcement**	**Post SDIL announcement**
Liable drinks				
Higher tier (≥8 g sugar per 100 ml)	913 (144)	652 (152)	101.4 (15.8)	72.4 (16.6)
Lower tier (≥5 g to <8 g sugar per 100 ml)	163 (39)	140 (42)	10.6 (2.6)	9.2 (2.7)
No levy (<5 g sugar per 100 ml)	2,461 (229)	2,520 (293)	12.1 (1.7)	12.0 (2.5)
>0 g to <5 g sugar per 100 ml	809 (84)	741 (96)	12.1 (1.7)	12.0 (2.5)
0 g sugar per 100 ml	1,053 (114)	1,076 (145)	0 (0)	0 (0)
Bottled water	599 (77)	703 (89)	0 (0)	0 (0)
Exempt drinks				
Alcoholic drinks	1,925 (443)	1,836 (509)	-	-
Milk and milk-based drinks	3,662 (203)	3,414 (224)	178.1 (9.9)	165.7 (11.1)
No-added-sugar fruit juices	529 (37)	488 (51)	52.1 (3.8)	47.2 (4.9)
Drinks sold as powders (g)	98 (12)	85 (11)	21.2 (3.4)	18.0 (3.2)
Confectionery (g)	318 (96)	294 (92)	178.9 (53.9)	164.7 (51.8)
Toiletries	124 (10)	118 (10)	-	-

Abbreviation: SDIL, Soft Drinks Industry Levy.

https://doi.org/10.1371/journal.pmed.1003269.t001

counterfactual at the first week after the SDIL announcement controlling for the underlying trends in household purchases through purchases of toiletries. The trend change is the mean change in the slope of purchases following the announcement. The absolute and relative differences represent the difference between the counterfactual and the model estimates in the final week of the study. Results are displayed graphically in Figs 1 and 2 for levy-eligible drinks and confectionery (and in S2 Text for other drink categories). The dashed line (counterfactual)

Table 2. Adjusted change in mean volume of drinks and confectionery purchased per household per week (95% CI) (level) and adjusted change per week (trend) post announcement of the Soft Drinks Industry Levy, including toiletries as a control condition, with absolute and relative differences in purchased volume at 2 years post announcement.

			Change at 2 years post announcement	
**Category**	**Level change (ml/g)**	**Trend change (ml/g per week)**	**Absolute change (ml/g)**	**Relative change (%)**
Liable drinks				
Higher tier (≥8 g sugar per 100 ml)	**60.5 (14.5–106.5)**	−0.4 (−1.2 to 0.3)	16.5 (−72.3 to 105.2)	3.5 (−16.2 to 23.3)
Lower tier (≥5 g to <8 g sugar per 100 ml)†	−17.5 (−43.0 to 8.0)	**−0.01 (−0.02 to −0.004)**	**−96.1 (−144.2 to −48.0)**	**−53.0 (−68.0 to −38.0)**
No levy (<5 g sugar per 100 ml)	−11.2 (−159.3 to 137.0)	−0.4 (−3.0 to 2.1)	−58.3 (−362.3 to 245.7)	−2.2 (−13.7 to 9.2)
Drinks with >0 g to <5 g sugar per 100 ml	−16.0 (−62.1 to 30.1)	0.4 (−0.4 to 1.2)	26.8 (−66.1 to 119.6)	3.8 (−9.9 to 17.6)
Drinks with 0 g sugar per 100 ml	−0.7 (−73.9 to 72.6)	0.02 (−1.4 to 1.4)	1.0 (−163.5 to 165.5)	0.1 (−14.9 to 15.1)
Bottled water	13.4 (−36.9 to 63.8)	−0.7 (−1.6 to 0.2)	−54.9 (−160.7 to 50.8)	−7.0 (−19.6 to 5.6)
Exempt drinks				
Alcoholic drinks	−12.4 (−147.3 to 122.4)	−0.8 (−2.9 to 1.3)	−99.0 (−356.6 to 158.5)	−5.3 (−18.5 to 7.8)
Milk and milk-based drinks	−59.8 (−160.7 to 41.1)	**−2.5 (−4.2 to −0.7)**	**−317.5 (−521.5 to −113.4)**	**−8.9 (−14.1 to −3.6)**
No-added-sugar fruit juices	6.3 (−17.8 to 34.0)	**0.01 (0.01–0.02)**	14.8 (−27.6 to 57.3)	3.0 (−5.9 to 12.0)
Drinks sold as a powder (g)	−1.7 (−11.2 to 7.8)	−0.05 (−0.2 to 0.1)	−6.6 (−25.4 to 12.2)	−7.8 (−28.5 to 12.9)
Confectionery (g)	−43.8 (−125.5 to 37.9)	−0.05 (−1.6 to 1.5)	−48.5 (−230.5 to 133.5)	−13.8 (−59.0 to 31.4)

Estimates statistically significant at the $p < 0.05$ level are highlighted in bold.

† Trend^2 indicates the volume change multiplied by weeks since the announcement, squared.

https://doi.org/10.1371/journal.pmed.1003269.t002

Table 3. Adjusted change in mean sugar in drinks and confectionery purchased per household per week (95% CI) (level) and adjusted change per week (trend) post announcement of the Soft Drinks Industry Levy, including toiletries as a control condition, with absolute and relative differences in purchased volume at 2 years post announcement.

			Change at 2 years post announcement	
**Category**	**Level change (g)**	**Trend change (g per week)**	**Absolute change (g)**	**Relative change (%)**
Liable drinks				
Higher tier (≥8 g sugar per 100 ml)	**9.2 (2.1–16.3)**	0.01 (−0.1 to 0.1)	10.5 (−3.1 to 24.2)	24.6 (−14.8 to 64.0)
Lower tier (≥5 g to <8 g sugar per 100 ml)†	−1.1 (−2.9 to 0.6)	**−0.001 (−0.001 to −0.0002)**	**−6.4 (−9.8 to −3.1)**	**−53.1 (−69.0 to −37.3)**
No levy (<5 g sugar per 100 ml)†	−0.3 (−1.5 to 0.9)	**0.0005 (0.0002–0.001)**	**6.1 (3.9–8.2)**	**68.8 (19.1–118.6)**
Drinks with >0 g to <5 g sugar per 100 ml†	−0.4 (−1.6 to 0.9)	**0.0005 (0.0001–0.001)**	**6.0 (3.8–8.1)**	**66.8 (18.9–114.6)**
Exempt drinks				
Milk and milk-based drinks†	−0.9 (−9.4 to 7.6)	−0.002 (−0.004 to 0.001)	−7.5 (−23.4 to 8.3)	−4.7 (−13.6 to 4.3)
No-added-sugar fruit juices	−1.5 (−4.3 to 1.3)	0.04 (−0.01 to 0.1)	2.2 (−3.5 to 7.9)	4.9 (−8.6 to 18.4)
Drinks sold as a powder (g)	−0.8 (−3.3 to 1.8)	0.003 (−0.04 to 0.1)	−0.5 (−6.0 to 5.1)	−2.6 (−33.9 to 28.6)
Confectionery	−26.1 (−73.1 to 21.0)	−0.01 (−1.0 to 1.0)	−26.8 (−140.9 to 87.4)	−13.6 (−62.6 to 35.4)

Estimates statistically significant at the $p < 0.05$ level are highlighted in bold; drinks with 0 g sugar per 100 ml and bottled water are excluded, as they contain no sugar; alcoholic drinks are excluded, as no information on sugar content was available.

†Trend^2 indicates the volume change multiplied by weeks since the announcement, squared.

https://doi.org/10.1371/journal.pmed.1003269.t003

displays a continuation of the observed preannouncement trend controlled for changes captured by toiletries. The impact of the announcement of the SDIL upon purchasing can be observed by comparing the solid to the dashed lines. Each combination of drinks category or confectionery with control was estimated and adjusted for autocorrelation distinctly; as a result, the plotted toiletries estimates may vary between combinations.

In the 2 years preannouncement, there was a marked decline in household purchasing of drinks in the higher levy tier, with some increase in purchasing of drinks in the lower levy tier and confectionery. Similar trends were seen in total sugar purchased from these categories.

The announcement of the SDIL was associated with a significant level increase in household weekly purchase volume (p-value < 0.001) and the amount of sugar (p-value = 0.011) from drinks in the higher levy tier. However, there was no change in trend of either outcome. At 2 years post announcement, there was no difference in volume of or sugar from drinks in the higher levy tier purchased per household per week compared to the counterfactual of no announcement.

In contrast, the SDIL announcement was associated with a reversal of the existing upward trend in volume of and amount of sugar in household purchases of drinks in the lower levy tier. These changes led to a −53.0% (95% CI −68.0 to −38.0) fall in the volume of and a −53.1% (95% CI −69.0 to −37.3) fall in the amount of sugar from drinks in purchases of lower-levy-tier drinks per household per week at 2 years post announcement (equivalent to a −96.1 ml [95% CI −144.2 to −48.0] reduction in volume and −6.4-g [95% CI −9.8 to −3.1] reduction in sugar purchased in these drinks per household per week).

Whereas the announcement of the SDIL was not associated with a level or trend change in volume of household purchases of no-levy drinks, there was a reversal of the existing downward trend in the amount of sugar in household purchases of these drinks. At 2 years post announcement, these changes led to a 68.8% (95% CI 19.1 to 118.6) increase in the amount of sugar in purchases of no-levy drinks per household per week but no change in volume purchased (equivalent to a 6.1-g [95% CI 3.9 to 8.2] increase in sugar purchased in these drinks per household per week). This change appeared to be driven by changes in volume of and sugar in purchases of drinks containing >0 g but <5 g total sugar per 100 ml, with the

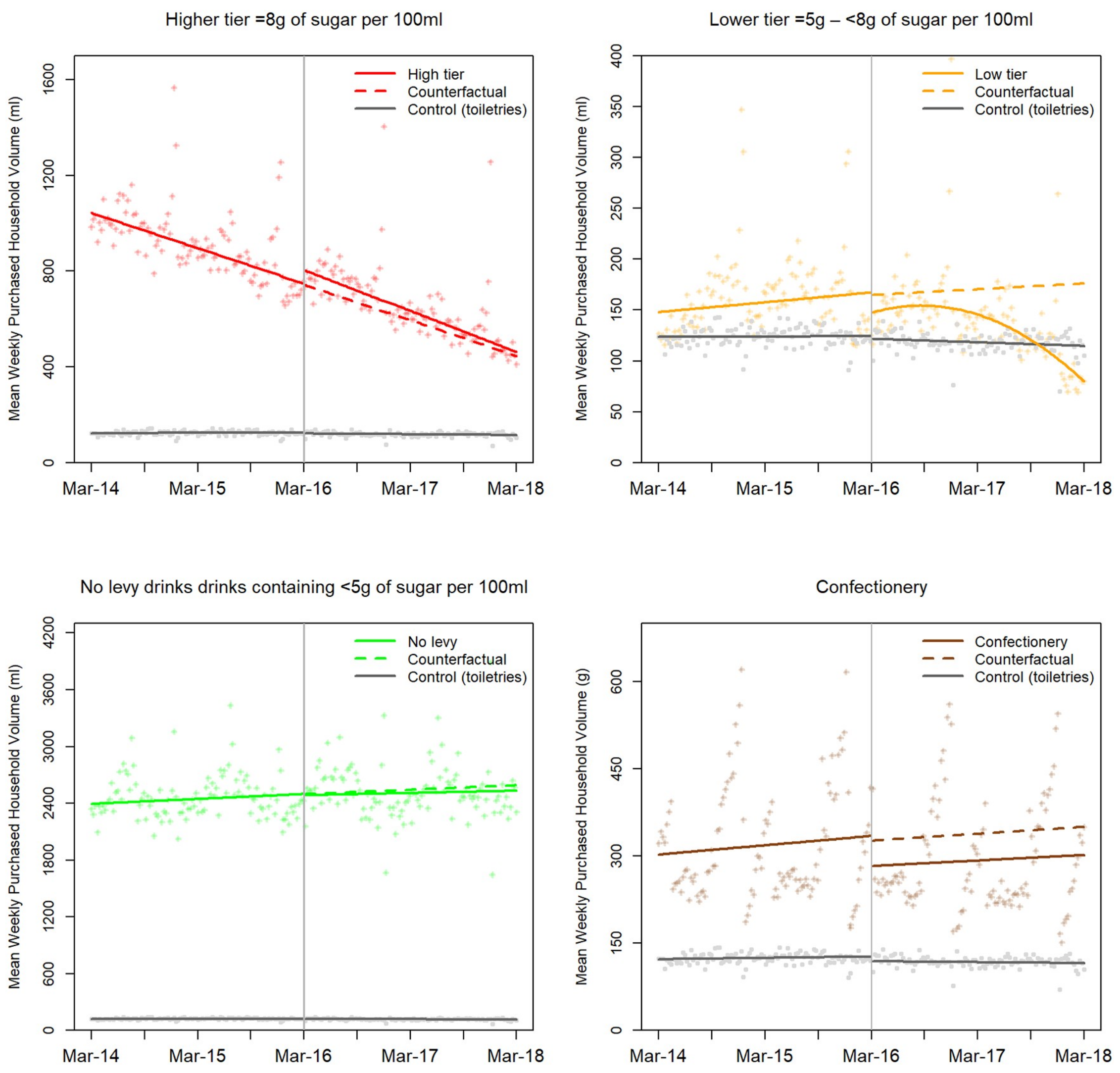

Fig 1. Observed and modelled volume of drinks liable to the Soft Drinks Industry Levy and weight of confectionery purchased per household per week, March 2014 to March 2018. Points are observed data, and coloured lines are modelled data; the vertical line indicates the point of announcement; y-axes vary between panels to maximise the resolution of figures; modelled purchases are presented as straight lines but include all adjustments as described in the Methods section; modelled postannouncement counterfactual lines may not be contiguous with modelled preannouncement lines because of the effects of controlling for toiletries.

https://doi.org/10.1371/journal.pmed.1003269.g001

difference between the fall in sugar from lower-tier drinks and the increase in sugar in no-levy drinks due to the removal of some sugar by manufacturers, meaning drinks shifted from being liable for the levy to not being liable.

There was no evidence that the announcement of the SDIL was associated with changes in weight of or amount of sugar in confectionery purchased per household per week. The same was true of alcoholic drinks and most other exempt drinks. The only exception was a −8.9% (95% CI −14.1 to −3.6) reduction in the volume of milk-based drinks at 2 years post announcement (equivalent to −317.5 ml [95% CI −521.5 to −113.4] less per household per week).

Excluding manufacturers with sales of <1 million litres or <0.5 million litres per year did not change the direction of any of these results (see S3 Text). Similarly, applying the SDIL thresholds of ≥8 g and of >5 g to <8 g sugar per 100 ml to group all drinks, including those

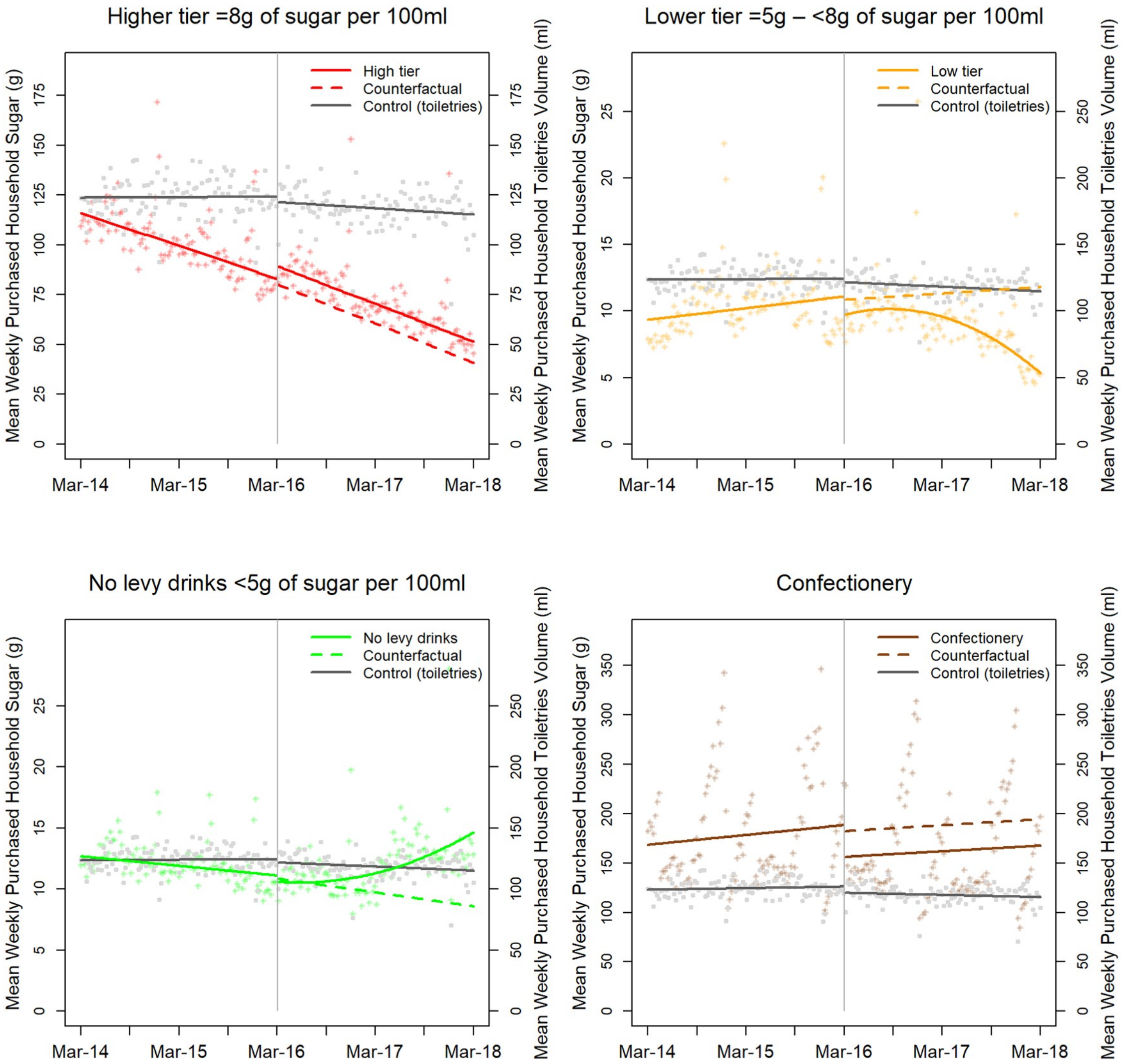

Fig 2. Observed and modelled amount of sugar in drinks liable to the Soft Drinks Industry Levy and confectionery purchased per household per week, March 2014 to March 2018. Points are observed data, and coloured lines are modelled data; the vertical line indicates the point of announcement; y-axes vary between panels to maximise the resolution of figures; modelled purchases are presented as straight lines but include all adjustments as described in the Methods section; modelled postannouncement counterfactual lines may not be contiguous with modelled preannouncement lines because of the effects of controlling for toiletries.

https://doi.org/10.1371/journal.pmed.1003269.g002

exempt from the levy, did not alter the results of the main analysis (see S4 Text). When drinks from all of the nonalcoholic drinks categories were combined, there was no evidence of a change in the volume of or amount of sugar in household drinks purchases as a result of the announcement of the levy at 2 years post announcement (see S4 Text).

Overall, results were similar in both the models with and without toiletries acting as a control condition, though the effect sizes did change. The trend in purchased volume of powdered drinks did reach statistical significance in the uncontrolled cases (−0.12 [95% CI −0.23 to −0.0002]), and sugar in confectionery did differ significantly following the announcement in the uncontrolled case (−44.4 [95% CI −85.5 to −3.3)]. The results of this are presented in S5 Text (S6 Table and S7 Table), which show model coefficients for the level and trend following the announcement of the SDIL together with 95% CIs.

Discussion

Summary of findings

We theorised that the announcement of the SDIL might lead to anticipatory changes, both by industry (as intended by government) and by consumers (via changes in consumer awareness, attitudes, or beliefs). Two years after the announcement, immediately prior to implementation of the SDIL when all drinks were combined, irrespective of levy eligibility or sugar content, we found no statistically significant change in the volume of (p-value = 0.07) or amount sugar in (p-value = 0.6) purchased drinks, indicating that additional action (including implementation of the SDIL) will be required to achieve a positive public health impact.

When we disaggregate levy-eligible drinks by category, we found no evidence of a change in volume of or the amount of sugar in household purchases of higher-levy-tier drinks compared to the counterfactual. This was against a backdrop of a substantial downward trend in purchasing of these drinks. Alongside, we found evidence of a 54% decrease in both the volume of and the amount of sugar in household purchases of lower-levy-tier drinks (equivalent to a reduction of 95 ml and 6 g of sugar in these drinks per household per week). There was no evidence of a change in the volume of household purchases of no-levy drinks with <5 g sugar per 100 ml. However, the reduction in sugar purchased from lower-levy-tier drinks was offset by a 78% increase in the amount of sugar purchased in no-levy-tier drinks (equivalent to an increase of 6 g of sugar in these drinks per household per week). We found no changes in purchasing of confectionery or alcoholic drinks associated with the announcement compared to the counterfactual scenario, suggesting that substitution to these categories did not occur. These results are consistent with either or both reformulation and introduction of new products, as well as consumer changes in purchasing. These findings help inform political discussions about rescinding sugar taxes (which has happened in, for example, Catalonia, Denmark, and Cook County, Illinois, and is not therefore merely a theoretical concern). It should also help countries implementing similar taxes to understand the timeline they might expect for effects to occur (and hence when evaluations should take place and when any impact on health outcomes may be observed).

Comparison of findings to previous research

Few previous studies have specifically explored the effects of the announcement of an SSB tax. In 2014, Chile changed the taxing structure of SSBs. A previous 13% ad valorem tax was reduced to 10% on drinks with <6.25 g sugar per 100 ml and increased to 18% for drinks with ≥6.25 g sugar per 100 ml. Drinks with no sugar, colouring, or flavouring remained untaxed. In line with the current findings, announcement of the tax 6 months prior to implementation was associated with an anticipatory decline in purchasing of drinks in the low, but not high, tax group [27,40].

A recent ITS of drinks available in supermarkets, rather than drinks purchased, in the UK found that the announcement of the SDIL was associated with a 20% drop in the proportion of levy-eligible drinks containing greater than 5 g of sugar per 100 ml (the minimum sugar threshold for the lower levy tier) [26]. Scarborough and colleagues also found evidence of 'strategic reformulation' with a new peak in the distribution of sugar content in drinks just below 5 g per 100 ml that was not previously evidenced. This is consistent with our finding of reductions in purchasing of lower-levy-tier drinks and increases in the sugar in but not volume of no-levy-tier drinks.

Similar to our unadjusted analysis (Table 1), a simple before-after analysis found that sales of levy-eligible soft drinks fell by 50% from 2015 to 2018, leading to an overall reduction in the amount of sugar in purchased soft drinks of 30% [27].

The 2018 annual report of the British Soft Drinks Association describes annual sales from 2012 to 2017 of drinks in a range of categories [44] (data source not specified). According to these figures, mean annual sales of bottled water increased by 16% between 2014–2015 and 2016–2017 whereas those of no-added-sugar fruit juice decreased by 6%. Comparable figures from our unadjusted analyses (Table 1) are 17% and 7%. The report does not provide annual sales for the other categories used here.

Data in a report by Public Health England exploring effects of their sugar reduction strategy on purchases in 2015 (before announcement of the SDIL) and 2017 (after announcement but before implementation) report a decrease in sales of higher- and lower-levy-tier drinks but an increase in sales of no-levy drinks [45]. We find the same direction of effect in our unadjusted analyses (Table 1).

Interpretation of findings

We found that the announcement of the SDIL was associated with a marked reduction in the volume of (p-value < 0.001) and sugar in (p-value < 0.001) drinks purchased in the lower levy tier, which accelerated as the date of levy implementation approached. Alongside, we saw an increase in the amount of sugar purchased in no-levy drinks (p-value < 0.001), which also accelerated as the date of levy implementation approached. We hypothesise that these results reflect reformulation of many drinks to just below the maximum sugar content for the no-levy tier (<5 g of sugar per 100 ml) and that this led to an overall increase in the average sugar content of drinks in this category. Prior to the levy announcement, this category was largely populated with zero-sugar drinks, but after implementation, a substantial new group of drinks with between 4.5 g and 4.9 g of sugar was seen [26].

We are not able to disentangle potential mechanisms of the changes in household purchasing associated with announcement of the SDIL reported here. Alongside reformulation, media coverage of the announcement and adaptive behaviours by industry, such as changes in marketing strategies, may have had an impact on purchasing by heightening awareness of the health harms of SSBs. For example, in another study, Mexicans who were aware of their SSB tax were more likely to report a recent decrease in consumption [20]. Although we found a small increase in household purchasing of drinks in the high levy tier immediately following the announcement of the SDIL, this did not result in a significant change in the purchased volume of or amount of sugar purchased in higher-levy-tier drinks at 2 years post announcement. It is possible that the small increase immediately following the announcement reflected stockpiling [46] in anticipation of price or recipe changes.

Despite no overall change in purchasing of drinks in the higher levy tier associated with the announcement of the SDIL, there was a marked existing downward trend in purchasing of these drinks, with purchased volume declining by 29% during the study period. The higher-levy-tier category is dominated by market-leading cola drinks, many of which have been reported as unlikely to reformulate [47]. Consumers of these products tend to have strong brand loyalty and are more likely to consume large volumes [48]. Any reformulation of drinks in this category may have been limited to products with small market shares. It is also possible that the large existing downward trend in purchasing represents the fastest that the market and consumers are able to change, with no additional impacts feasible from the announcement of the SDIL.

Potential unintended consequences of the SDIL include substitutions to other less healthful categories such as confectionery and alcoholic drinks. We had initially hypothesised that

households would maintain sugar levels by switching from SSBs to confectionery [34,35,49]. However, we did not find evidence of this, with a small though not significant reduction in weight of (p-value = 0.6) and sugar in (p-value = 0.7) household purchases of confectionery observed. We also found no evidence that the SDIL announcement was associated with changes in purchases of alcohol compared to the counterfactual scenario (p-value = 0.7). Changes in purchasing of confectionery and alcohol may still follow SDIL implementation, and this should be monitored.

Strengths and weaknesses of methods

This work was carried out using nationally representative household panel purchase data. However, the KWP data used only capture purchases brought home. Although KWP has recently established a smaller 'out-of-home' panel, data are not available prior to June 2015 and are only available from a smaller subpanel. It is possible that out-of-home purchasers responded differently to the announcement of the SDIL to those brought in to the home. However, the unadjusted changes in bottled water and no-sugar-added fruit juice reported here compare favourably to similar data on the full UK market, indicating that our results may be generalisable to all purchasing.

Household purchases do not necessarily equate to individual consumption, as drinks purchased may be wasted or shared unevenly within households. Nevertheless, purchases brought into the home as captured by KWP appear to provide a reasonably accurate estimate of consumption [50]. An alternative approach would have been longitudinal analyses of individual-household data. However, KWP is a consistently shifting panel of households who join and leave or are temporarily dropped owing to poor-quality data. Further, because of data cost, we only have data on the categories here—drinks, toiletries, and confectionery. Thus, when a household recorded purchases of drinks in earlier but not later weeks, we were unable to distinguish between the household having withdrawn from the panel, stopped purchasing soft drinks, or been excluded owing to poor data quality, precluding an analysis at the individual-household level.

We used KWP data on sugar content of purchased drinks. Nutritional data on existing products are checked by KWP every 6 months. This may lead to a lag between reformulation and changes in KWP data, making it possible that we have underestimated changes in purchasing of sugar associated with reformulation. However, our validity checks of KWP sugar content data found no indication of systematic differences between KWP sugar content data and contemporaneous values listed on supermarket websites (S6 Text).

Attribution of effects in an ITS analysis is vulnerable to other interventions with the potential to impact on the outcomes of interest occurring at, or near, the same time. The announcement of the SDIL was part of the UK Chancellor's 2016 budget speech. This contained other announcements that may have impacted on household purchases. The inclusion of a control category (toiletries) attempted to take any underlying changes in household purchasing into account. However, toiletries were selected before the data were purchased; thus, they may be subject to trends that lessen (or magnify) the effects reported here, and a more appropriate group of products to function as a control may exist.

The SDIL can be seen as part of a wider dialogue surrounding sugar and SSB consumption in the UK that includes the Scientific Advisory Committee on Nutrition's report on carbohydrates and health, the government's ongoing childhood obesity plan (with chapters 1 and 2 published in 2016 and 2018, respectively), and sugar- and calorie-reduction strategies (published in 2017 and 2018, respectively) [4,51–54]. Publication of all of these, in addition to television documentaries such as *Jamie's Sugar Rush* [55] and *That Sugar Film* [56], may have played some part in the changes reported here.

We took a hypothesis-driven approach to focus our analysis on the point of announcement of the SDIL. A data-driven search for other inflexion points may have revealed different patterns, but we felt this would have been conceptually confusing and potentially difficult to interpret. Future work could explore this further.

Panel data can be limited by changes in panel composition over time. However, the demographic characteristics of the KWP panel remained similar over the study period. Furthermore, proprietary weightings provided by KWP and used throughout account for nonconsumers and adjust for variations in panel composition. Resource constraints limited us from studying impacts across all food and drink categories. Our analyses focus on the impact of the SDIL announcement on purchasing. Further work will be required to determine the impact of implementation on purchases and relevant health outcomes.

Implications of findings

Our findings indicate that the announcement of the SDIL was associated with changes in purchasing of soft drinks in a number of categories that may be the result of product reformulation, changes in consumer awareness, attitudes and beliefs, or a combination of both. Overall, however, we found no change in total volume of or sugar in household purchases of all soft drinks combined, indicating that further action will be required beyond the announcement of the levy to achieve a positive public health impact—including the levy's actual implementation. The announcement of an SSB tax is not an isolated intervention that can likely be replicated in other contexts, as the reaction to the announcement occurs on the assumption that the tax will be implemented. Therefore, the announcement of an SSB tax on its own, whilst making it clear that it would not be implemented, is unlikely to produce the same findings as those reported here. Nevertheless, in line with recent developments in the public health literature [25], by theorising the SDIL as a series of events in a complex system, with the announcement as a key early event leading to changes in anticipation of implementation, our findings help to build a complete picture of the impacts of the SDIL, when these occurred, and via what mechanisms.

Conclusions

The announcement of the UK SDIL was associated with a 96.1 ml decrease in the volume of household purchases of lower-levy-tier drinks containing $\geq$5 to $<$8 g sugar per 100 ml per week compared to the counterfactual estimated from preannouncement trends, equating to 6.4 g less sugar per household per week. No change in the substantial existing downward trends in volume of and amount of sugar in higher-levy-tier drinks with $>$8 g sugar per 100 ml were seen. However, a 6.1 g increase in sugar purchased from drinks in the no-levy tier was seen and was isolated to the subcategory of drinks with $>$0 g but $<$5 g sugar per 100 ml. There was no evidence of substitution to alcoholic drinks or confectionery. These findings may reflect reformulation of drinks from the low levy to no-levy tier with removal of some—but not all—sugar, alongside changes in consumer attitudes and beliefs. Any reformulation of drinks in the higher levy tier may have been limited to drinks with small market shares. Overall there was no change in total volume of or sugar in household purchases of all soft drinks combined, indicating that further action, including implementation of the SDIL, will be required to achieve public health impact. Future work should determine the impacts of the implementation of the SDIL.

Supporting information

S1 Checklist. STROBE checklist of item that should be included in reports of cohort studies. STROBE, Strengthening the Reporting of Observational Studies in Epidemiology.
(DOCX)

S1 Fig. Observed and modelled volume of drinks liable to the Soft Drinks Industry Levy and weight of confectionery purchased per household per week, March 2014 to March 2018. Points are observed data, and coloured lines are modelled data; the vertical line indicates the point of announcement; y-axes vary between panels to maximise the resolution of figures; modelled purchases are presented as straight lines but include all adjustments as described in the Methods section; modelled postannouncement counterfactual lines may not be contiguous with modelled preannouncement lines because of the effects of controlling for toiletries.
(TIF)

S2 Fig. Observed and modelled amount of sugar in drinks liable to the Soft Drinks Industry Levy and confectionery purchased per household per week, March 2014 to March 2018. Points are observed data, and coloured lines are modelled data; the vertical line indicates the point of announcement; y-axes vary between panels to maximise the resolution of figures; modelled purchases are presented as straight lines but include all adjustments as described in the Methods section; modelled postannouncement counterfactual lines may not be contiguous with modelled preannouncement lines because of the effects of controlling for toiletries.
(TIF)

S3 Fig. Agreement between nutritional information reported by KWP and that collected from archived and current supermarket and manufacturer data collected by Archive.org. KWP, Kantar Worldpanel.
(TIF)

S1 Text. Demographic characteristics of Kantar Worldpanel households from March 2014 to March 2018 (weighted).
(DOCX)

S2 Text. Additional drinks categories.
(DOCX)

S3 Text. Sensitivity analysis 1: Exemptions for small manufacturers and producers.
(DOCX)

S4 Text. Sensitivity analysis 2: Combining drinks categories.
(DOCX)

S5 Text. Sensitivity analysis 3: Uncontrolled interrupted time series analysis.
(DOCX)

S6 Text. Validity checks of data on sugar content of drinks.
(DOCX)

Acknowledgments

The views expressed are those of the authors and not necessarily those of the National Health Service, the NIHR, or the Department of Health and Social Care, UK.

Author Contributions

Conceptualization: Tarra L. Penney, Oliver Mytton, Adam Briggs, Steven Cummins, Mike Rayner, Harry Rutter, Peter Scarborough, Richard D. Smith, Martin White, Jean Adams.

Data curation: David Pell, Stephen J. Sharp.

Formal analysis: David Pell, Stephen J. Sharp.

Funding acquisition: Tarra L. Penney, Oliver Mytton, Adam Briggs, Steven Cummins, Mike Rayner, Harry Rutter, Peter Scarborough, Richard D. Smith, Martin White, Jean Adams.

Investigation: David Pell, Oliver Mytton, Jean Adams.

Methodology: David Pell.

Project administration: Tarra L. Penney.

Supervision: Jean Adams.

Validation: David Pell.

Visualization: David Pell, Oliver Mytton.

Writing – original draft: David Pell, Jean Adams.

Writing – review & editing: David Pell, Tarra L. Penney, Oliver Mytton, Adam Briggs, Steven Cummins, Mike Rayner, Harry Rutter, Peter Scarborough, Stephen J. Sharp, Richard D. Smith, Martin White, Jean Adams.

References

1. Moynihan PJ, Kelly SAM. Effect on Caries of Restricting Sugars Intake: Systematic Review to Inform WHO Guidelines. J Dent Res. 2014; 93:8–18. https://doi.org/10.1177/0022034513508954. PMID: 24323509
2. Imamura F, O'Connor L, Ye Z, Mursu J, Hayashino Y, Bhupathiraju SN, et al. Consumption of sugar sweetened beverages, artificially sweetened beverages, and fruit juice and incidence of type 2 diabetes: systematic review, meta-analysis, and estimation of population attributable fraction. Br J Sports Med. 2016; 50:496–504. https://doi.org/10.1136/bjsports-2016-h3576rep. PMID: 27044603
3. Xi B, Huang Y, Reilly KH, Li S, Zheng R, Barrio-Lopez MT, et al. Sugar-sweetened beverages and risk of hypertension and CVD: a dose–response meta-analysis. Br J Nutr. 2015; 113:709–17. https://doi.org/10.1017/S0007114514004383. PMID: 25735740
4. Tedstone A, Targett V, Allen R. Sugar reduction: the evidence for action. 2015. PHE publications gateway number 2015391.
5. Morgan E, Dent M. The economic burden of obesity. Oxford: National Obesity Observatory; 2010.
6. World Health Organization. Consideration of the evidence on childhood obesity for the Commission on Ending Childhood Obesity: report of the ad hoc working group on science and evidence for ending childhood obesity. Geneva, Switzerland: WHO; 2016.
7. World Health Organization. Tackling NCDs: "Best Buys" and Other Recommended Interventions for the Prevention and Control of Noncommunicable Diseases. Geneva, Switzerland: WHO; 2017.
8. Dharmasena S, Capps O. Intended and unintended consequences of a proposed national tax on sugar-sweetened beverages to combat the U.S. obesity problem. Health Econ. 2012; 21:669–94. https://doi.org/10.1002/hec.1738. PMID: 21538676
9. Falbe J, Rojas N, Grummon AH, Madsen KA. Higher Retail Prices of Sugar-Sweetened Beverages 3 Months After Implementation of an Excise Tax in Berkeley, California. Am J Public Health. 2015; 105:2194–201. https://doi.org/10.2105/AJPH.2015.302881. PMID: 26444622
10. Falbe J, Thompson HR, Becker CM, Rojas N, McCulloch CE, Madsen KA. Impact of the Berkeley Excise Tax on Sugar-Sweetened Beverage Consumption. Am J Public Health. 2016; 106:1865–71. https://doi.org/10.2105/AJPH.2016.303362. PMID: 27552267
11. Batis C, Rivera JA, Popkin BM, Taillie LS. First-Year Evaluation of Mexico's Tax on Nonessential Energy-Dense Foods: An Observational Study. PLoS Med. 2016; 13. https://doi.org/10.1371/journal.pmed.1002057. PMID: 27379797
12. Colchero MA, Rivera-Dommarco J, Popkin BM, Ng SW. In Mexico, Evidence Of Sustained Consumer Response Two Years After Implementing A Sugar-Sweetened Beverage Tax. Health Aff (Millwood). 2017; 36:564–71. https://doi.org/10.1377/hlthaff.2016.1231. PMID: 28228484
13. Code général des impôts, Article 1613 (2020).
14. World Health Organization. Taking action on childhood obesity report. WHO. 2018 [cited 2019 Apr 17]. http://www.who.int/end-childhood-obesity/publications/taking-action-childhood-obesity-report/en/

15. Silver LD, Ng SW, Ryan-Ibarra S, Taillie LS, Induni M, Miles DR, et al. Changes in prices, sales, consumer spending, and beverage consumption one year after a tax on sugar-sweetened beverages in Berkeley, California, US: A before-and-after study. PLoS Med. 2017; 14:e1002283. https://doi.org/10.1371/journal.pmed.1002283. PMID: 28419108

16. Colchero MA, Popkin BM, Rivera JA, Ng SW. Beverage purchases from stores in Mexico under the excise tax on sugar sweetened beverages: observational study. BMJ. 2016; 352:h6704. https://doi.org/10.1136/bmj.h6704. PMID: 26738745

17. Teng AM, Jones AC, Mizdrak A, Signal L, Genç M, Wilson N. Impact of sugar-sweetened beverage taxes on purchases and dietary intake: Systematic review and meta-analysis. Obes Rev. 2019; 20:1187–204. https://doi.org/10.1111/obr.12868. PMID: 31218808

18. Alvarado M, Kostova D, Suhrcke M, Hambleton I, Hassell T, Samuels TA, et al. Trends in beverage prices following the introduction of a tax on sugar-sweetened beverages in Barbados. Prev Med. 2017; 105:S23–5. https://doi.org/10.1016/j.ypmed.2017.07.013. PMID: 28716655

19. Cawley J, Willage B, Frisvold D. Pass-Through of a Tax on Sugar-Sweetened Beverages at the Philadelphia International Airport. JAMA. 2018; 319:305–6. https://doi.org/10.1001/jama.2017.16903. PMID: 29071342

20. Álvarez-Sánchez C, Contento I, Jiménez-Aguilar A, Koch P, Gray HL, Guerra LA, et al. Does the Mexican sugar-sweetened beverage tax have a signaling effect? ENSANUT 2016. PLoS ONE. 2018; 13: e0199337. https://doi.org/10.1371/journal.pone.0199337. PMID: 30133438

21. HM Revenue & Customs. Soft Drinks Industry Levy. GOVUK. 2016 Dec 5 [cited 2019 Jan 14]. https://www.gov.uk/government/publications/soft-drinks-industry-levy/soft-drinks-industry-levy

22. HM Revenue & Customs. Check if your drink is liable for the Soft Drinks Industry Levy. GOVUK. 2018 Apr 3 [cited 2018 Sep 18]. https://www.gov.uk/guidance/check-if-your-drink-is-liable-for-the-soft-drinks-industry-levy

23. An Roinn Airgeadais Department of Finance. Sugar-Sweetened Drinks Tax Public Consulation. An Roinn Airgeadais Department of Finance; 2016.

24. Economics Tax Analysis Chief Directorate. Taxation of sugar sweetened beverages. National Treasury Republic of South Africa; 2016.

25. Buckton CH, Patterson C, Hyseni L, Katikireddi SV, Lloyd-Williams F, Elliott-Green A, et al. The palatability of sugar-sweetened beverage taxation: A content analysis of newspaper coverage of the UK sugar debate. PLoS ONE. 2018; 13:e0207576. https://doi.org/10.1371/journal.pone.0207576. PMID: 30517133

26. Scarborough P, Adhikari V, Harrington RA, Elhussein A, Briggs A, Rayner M, et al. Impact of the announcement and implementation of the UK Soft Drinks Industry Levy on sugar content, price, product size and number of available soft drinks in the UK, 2015–19: A controlled interrupted time series analysis. PLoS Med. 2020; 17:e1003025. https://doi.org/10.1371/journal.pmed.1003025. PMID: 32045418

27. Bandy LK, Scarborough P, Harrington RA, Rayner M, Jebb SA. Reductions in sugar sales from soft drinks in the UK from 2015 to 2018. BMC Med. 2020; 18:20. https://doi.org/10.1186/s12916-019-1477-4. PMID: 31931800

28. Rutter H, Savona N, Glonti K, Bibby J, Cummins S, Finegood DT, et al. The need for a complex systems model of evidence for public health. Lancet Lond Engl. 2017; 390:2602–4. https://doi.org/10.1016/S0140-6736(17)31267-9. PMID: 28622953

29. White M. Evaluation of the health impacts of the UK Treasury Soft Drinks Industry Levy (SDIL). 2017. https://doi.org/10.1186/ISRCTN18042742

30. Penney TL. ISRCTN18042742: Evaluation of the health impacts of the UK Treasury Soft Drinks Industry Levy (SDIL). 2017. https://doi.org/10.1186/ISRCTN18042742

31. Bernal JL, Cummins S, Gasparrini A. Interrupted time series regression for the evaluation of public health interventions: a tutorial. Int J Epidemiol. 2017; 46:348–55. https://doi.org/10.1093/ije/dyw098. PMID: 27283160

32. Penfold RB, Zhang F. Use of Interrupted Time Series Analysis in Evaluating Health Care Quality Improvements. Acad Pediatr. 2013; 13:S38–44. https://doi.org/10.1016/j.acap.2013.08.002. PMID: 24268083

33. Lopez Bernal J, Cummins S, Gasparrini A. The use of controls in interrupted time series studies of public health interventions. Int J Epidemiol. 2018; 47:2082–2093. https://doi.org/10.1093/ije/dyy135 PMID: 29982445

34. A Tax on Sodas? Becker. In: Becker-Posner Blog [Internet]. 2009 May 24 [cited 2020 Mar 5]. https://www.becker-posner-blog.com/2009/05/a-tax-on-sodas-becker.html

35. A Soda or Calorie Tax to Reduce Obesity–Posner. In: Becker-Posner Blog [Internet]. 2009 May 24 [cited 2020 Mar 5]. https://www.becker-posner-blog.com/2009/05/a-soda-or-calorie-tax-to-reduce-obesity–posner.html

36. Smith K, Griffith R, O'Connell M. Measuring the quality of people's diets: a comparison of intake and purchase data. Econometrics and IO of food and nutrition; Toulouse School of Economics; 2012.

37. Linden A. Conducting Interrupted Time-series Analysis for Single- and Multiple-group Comparisons. Stata J. 2015; 15:480–500. https://doi.org/10.1177/1536867X1501500208.

38. UK and regional series. Met Office. https://www.metoffice.gov.uk/climate/uk/summaries/datasets [cited 2018 Dec 6]

39. Zhang F, Wagner AK, Soumerai SB, Ross-Degnan D. Methods for estimating confidence intervals in interrupted time series analyses of health interventions. J Clin Epidemiol. 2009; 62:143–8. https://doi.org/10.1016/j.jclinepi.2008.08.007. PMID: 19010644

40. Families and households. Office for National Statistics. 2019 [2019 Mar 25]. https://www.ons.gov.uk/peoplepopulationandcommunity/birthsdeathsandmarriages/families/datasets/familiesandhouseholdsfamiliesandhouseholds

41. The effects of taxes and benefits on household income, disposable income estimate: 2018. Office for National Statistics. https://www.ons.gov.uk/peoplepopulationandcommunity/personalandhouseholdfinances/incomeandwealth/datasets/householddisposableincomeandinequality [cited 2019 Mar 25]

42. Qualifications in the population. GOVUK. 2014 Mar 5 [updated 2016 Nov 17; cited 2019 Mar 25]. https://www.gov.uk/government/statistical-data-sets/fe-data-library-qualifications-in-the-population-based-on-the-labour-force-survey

43. Social Grade. National Readership Survey. http://www.nrs.co.uk/nrs-print/lifestyle-and-classification-data/social-grade/ [cited 2019 May 21]

44. British Soft Drinks Association. 2018 Annual Report. 2019.

45. Public Health England. Sugar reduction: report on first year progress. 2018.

46. Irn Bru panic as fans stockpile drink. BBC. 2018 Jan 4 [2019 Jul 2]. https://www.bbc.com/news/uk-scotland-glasgow-west-42565363

47. Wood Z. Coca-Cola to sell smaller bottles at higher prices in response to sugar tax. The Guardian. 2018 Jan 5.

48. Do brands compete or coexist? How persistence of brand loyalty segments the market. Eur J Mark. 2018. https://doi.org/10.1108/EJM-07-2018-0489.

49. Smith RD, Cornelsen L, Quirmbach D, Jebb SA, Marteau TM. Are sweet snacks more sensitive to price increases than sugar-sweetened beverages: analysis of British food purchase data. BMJ Open. 2018; 8:e019788. https://doi.org/10.1136/bmjopen-2017-019788. PMID: 29700100

50. Appelhans BM, French SA, Tangney CC, Powell LM, Wang Y. To what extent do food purchases reflect shoppers' diet quality and nutrient intake? Int J Behav Nutr Phys Act. 2017; 14. https://doi.org/10.1186/s12966-017-0502-2. PMID: 28399887

51. Tedstone A, Anderson S, Allen R. Sugar reduction responding to the challenge. Public Health England; 2014.

52. Calorie reduction: the scope and ambition for action. GOVUK. 2018 Mar 6 [cited 2019 Jun 13]. https://www.gov.uk/government/publications/calorie-reduction-the-scope-and-ambition-for-action

53. Childhood obesity: a plan for action. GOVUK. 2017 Jan 20 [cited 2019 Jun 13]. https://www.gov.uk/government/publications/childhood-obesity-a-plan-for-action/childhood-obesity-a-plan-for-action

54. Scientific Advisory Committee on Nutrition. Carbohydrates and Health Report. TSO; 2015.

55. Cooper V, director. Jamie's Sugar Rush [Film]; 2015. Fresh One Productions.

56. Gameau D, director and writer. That Sugar Film [Film]; 2014. Richmond, Australia: Madman Production Company.
